# The Molecular Role of HIF1α Is Elucidated in Chronic Myeloid Leukemia

**DOI:** 10.3389/fonc.2022.912942

**Published:** 2022-06-30

**Authors:** Vivek Singh, Ranjana Singh, Rashmi Kushwaha, Shailendra Prasad Verma, Anil Kumar Tripathi, Abbas Ali Mahdi

**Affiliations:** ^1^ Department of Biochemistry, King George’s Medical University, Lucknow, India; ^2^ Department of Pathology, King George’s Medical University, Lucknow, India; ^3^ Department of Clinical Hematology, King George’s Medical University, Lucknow, India

**Keywords:** chronic myeloid leukemia (CML), hypoxia, redox system, BCR/ABL1 translocation, HIF1α, notch signaling pathway, Krebs metabolite

## Abstract

Chronic myeloid leukemia (CML) is potentially fatal blood cancer, but there is an unmet need to discover novel molecular biomarkers. The hypothesis of this study aimed to elucidate the relationship of HIF1α with the redox system, Krebs cycles, notch1, and other regulatory proteins to better understand the pathophysiology and clinical relevance in chronic myeloid leukemia (CML) patients, as the molecular mechanism of this axis is still not clear. This study included CML patient samples (n = 60; 60: blood; 10: bone marrow tissues) and compared them with healthy controls (n = 20; blood). Clinical diagnosis confirmed on bone marrow aspiration, marrow trephine biopsy, and BCR/ABL1 translocation. Cases were subclassified into chronic, accelerated, and blast crises as per WHO guidelines. Molecular experiments included redox parameters, DNA fragmentation, Krebs cycle metabolites, and gene expression by RT-PCR/Western blot/LC-MS, PPI (STRING), Pearson correlation, and ROC curve analysis. Here, our findings show that p210/p190BCR/ABL1 translocation is common in all blast crisis phases of CML. Redox factor/Krebs oncometabolite concentrations were high, leading to upregulation and stabilization of HIF1α. HIF1α leads to the pathogenesis in CML cells by upregulating their downstream genes (Notch 2/4/Ikaros/SIRT1/Foxo-3a/p53, etc.). Whereas, downregulated ubiquitin proteasomal and apoptotic factors in CML pateints, can trigger degradation of HIF1α through proline hydroxylation. However, HIF1α showed a negative corelation with the notch1 pathway. Notch1 plays a tumor-suppressive role in CML and might have the potential to be used as a diagnostic marker along with other factors in CML patients. The outcome also revealed that oxidant treatment could not be effective in augmentation with conventional therapy because CML cells can enhance the levels of antioxidants for their survival. HIF1α might be a novel therapeutic target other than BCR/ABL1 translocation.

## Introduction

Oxygen (O_2_) plays an important role in regulating metabolism and aerobic respiration as an electron acceptor in the electron transport system and is responsible for the production of ATP in the eukaryotic system (1). Therefore, a requisite amount of oxygen is always needed by the human body to maintain various kinds of physiological functions for survival. Whenever the oxygen requirement goes beyond its level of supply, the concentration of oxygen decreases in tissues (termed hypoxia), leading to a metabolic catastrophe and consequentially hampering biological functions and viability. ROS (reactive oxygen species) are secondary products of aerobic respiration. ROS are mainly presented as OH* (hydroxyl radicals), H_2_O_2_ (hydrogen peroxide), and O^2–^ (superoxide anion), all of which have intrinsic chemical behaviors and reactivity towards the whole physiological system. ROS are frequently linked with oxidative insult through lipid peroxidation, proteins, and DNA degradation (2). H_2_O_2_ is often derived from O^2–^ (superoxide) by NADPH oxidase (NOX) enzymes and mitochondria (3, 4). O^2–^ (superoxide) is generated from molecular oxygen (O_2_) by a mechanism of one-electron reduction and immediately converted by superoxide dismutase (SOD) into H_2_O_2_. SOD enzymes avert the aggregation of superoxide ions that can hamper and functionally latent proteins consisting of iron-sulfur clusters (5). On the other hand, reactive hydroxyl radicals can initiate lipid peroxidation, proteins, and DNA degradation, which consequentially destroys genomic integrity (6). Characteristically, OH* (hydroxyl radicals) are produced from hydrogen peroxide in the companionship of ferrous ions (Fenton reaction). In chronic myeloid leukemia cells (CML), the excessive degree of ROS formation is neutralized by the antioxidant activity of the cells, maintaining the balance of the redox system (7). If CML cells are not able to monitor their ROS production, then they are vulnerable to oxidative damage, resulting in anoikis (8, 9). Oxygen has a crucial role in normal physiological mechanisms, and human cells have evolved and formed well-organized oxygen sensing (HIF) domains (1). All O_2_ (oxygen)-related responses (hypoxia) are managed by HIFs (hypoxia-inducible factors) HIF exists as a heterodimer: a hypoxia-activated α subunit and a constitutively expressed β subunit, also known as aryl hydrocarbon nuclear receptor translocator (ARNT). There are three isoforms of the α subunit termed HIF-1α, HIF-2α and HIF-3α. HIF-1α and HIF-2α have been more extensively studied, whereas research on HIF-3α isoforms is relatively scarce. In general, HIF-2α regulates similar genes as HIF-1α, while HIF-3α acts a negative regulator of these genes (10, 11). In the last two decades, several works have enhanced our understanding of hypoxia at the cellular, biochemical and molecular levels. The responses at the cellular and developmental trajectories to hypoxia are mostly mediated by HIF1α, which is encoded by the HIF1α gene (12, 13). Aberrations and upregulation of the HIF1α gene are persuaded by genetic modifications, and hypoxia has been associated with various pathophysiologies of many diseases, including CML (14). Hypoxia-affected cells can stabilize a group of transcription factors associated with HIFs (hypoxia-inducible factors) (15). HIFs are heterodimer proteins comprising an oxygen-sensitive HIFα domain and a fundamentally inert HIFβ domain. The HIFα domain has been hydroxylated at proline sites by PHD (prolyl hydroxylase) enzymes, and these hydroxylated residues are then ubiquitinated by the E3 ubiquitin ligase along with pVHL (von Hippel-Lindau protein), which ultimately degrades HIFα protein in the proteasomal complex (16). However, in a hypoxic environment, the hydroxylation process is inhibited on HIFα, which ultimately inhibits HIFα degradation. However, HIFα is transported to the nucleus, where it forms dimers with HIFβ, regulates metabolic alterations to hypoxia, and induces the expression of angiogenic genes such as VEGF (vascular endothelial growth factor) (17). ROS production increases during hypoxia, stabilizes HIFα, and inhibits the function of PHDs (18, 19). The generation of ROS triggers HIFs, leading to pathogenesis in CML cells (20–24). HIF1α simultaneously persuades the transcription of multiple genes, which can participate in the pathogenesis (angiogenesis, anaerobic glycolytic metabolism, pH regulation, cancer metastasis, erythropoiesis, and cell proliferation and survival) of cancer cells (25, 26). HIF1α can also trigger inflammation and immunity by upregulating TNFα (tumor necrosis factor-α) and cancer metastasis by upregulating fibronectin-1 (27). Leukemia cells themselves enhance the level of ROS to switch on the peripheral signaling pathway, which establishes redox equilibrium. At the same time, CML cells also upregulate antioxidant activity to scavenge ROS, which induces oxidative stress and cell death. A combinatorial approach of decreasing oxygen levels and enhancing mitochondrial ROS (*m-ROS*) inhibits PHDs to upregulate HIF1α (18). *m-ROS* aggregation during normoxia was able to inhibit PHDs and activate HIF Krebs (TCA) cycle intermediates; succinate, fumarate, and malate can also inhibit PHDs during normoxia (16, 28). Succinate is an oncometabolite that has a multifaceted function at the cellular and molecular levels, and it accumulates because of mutation in the SDH enzyme that ultimately leads to a loss in functionality. Additionally, succinate also inhibits the function of PHDs, leading to the accumulation of HIF1α in the presence of oxygen (pseudohypoxia) (29). However, fumarate is a formidable suppressor of 2-OGDD enzymes (30, 31), so it can also retain the pseudohypoxic conditions that describe FH-deficient cancer cells by stabilizing HIF1α during normoxia by inhibiting the activity of PHD enzymes (32). Fumarate is an electrophile guide to succination in proteins, a phenomenon where fumarate binds and deactivates reactive thiol groups at cysteine residues (GAPDH, GMPR, etc.). This kind of protein alteration was first distinguished in diabetes, where the enhanced expression of fumarate leads to worsening β-cell function (33, 34). Hypoxia is also associated with cancer stemness in various tissue systems in the human body. The impact of HIFs on CSCs (cancer stem cells) is mostly induced through HIF-dependent stimulation of stem cell transcription factors. In addition to pluripotent components, epigenetic remodelers such as sirtuin-1 (SIRT-1) play vital roles in maintaining leukemia stem cell properties (35, 36), and they are always triggered during hypoxic conditions (37, 38). It has been widely described throughout previous work that cancer cells attaining epithelial-mesenchymal transition (EMT) acquire enhanced stem cell properties for self-renewal and copy for better pathogenesis (39). Hypoxia signaling can induce EMT through HIF1α-mediated activation of EIFs (EMT-inducing factors), including TWIST, SNAIL, and ZEB1 (40–42). In addition, hypoxia signaling can synchronize with various signaling pathways that induce stemness in CML cells. It has already been mentioned that HIF1α can regulate the TGFβ, Wnt, and Notch signaling pathways to promote the survival of leukemia cells (43–46).

In CML, Notch signalling has been demonstrated to mediate the disease progression and in K562 CML cell line model Notch signalling inhibited the development of erythroid/megakaryocytic cells by induction of *Hes1* and proliferation of K562 cells (44). Recently, Yang et al., showed that over-expression of *Notch2* inhibits the proliferation of CML cells. *Hes1* which is the most widely characterised Notch downstream target gene has been shown to immortalize committed progenitors and play a role in transformation of chronic-phase CML to blast crisis (45). However, the underlying molecular relationship between Notch signalling and CML remains largely unknown. Notch also has a role in mediating the cellular response to hypoxia through crosstalk with hypoxia-inducible factors 1- and 2-α (HIF1α, HIF2α). Inhibiting Notch signaling has been suggested as a therapeutic approach to prevent hypoxia-induced tumor invasion in uveal melanoma (16). In the same study, hypoxia was found to increase the levels of Notch ligands, including Jagged1. Blocking Notch signaling could thus be a promising approach to reduce hypoxia-mediated stress and thereby pro-angiogenic signaling, both suggested as driving factors in wet AMD. A recent study of combined targeting of HIF1α and VEGF in a hypoxia-driven model of retinal neovascularization concluded on beneficial effects of combinatory treatment. This raises the hypothesis that the interplay with the hypoxic pathways may also enable Notch blockade to increase the efficacy of the current VEGF-based treatment regimens in neovascular eye diseases. Hypoxia has also been found to up-regulate Notch3 in lung tissue, alluding to the hypothesis of Notch3 serving as a potential therapeutic target to reduce pulmonary arterial hypertension. Interestingly, Notch3 has also been shown to be crucial in pathological neovascularization in an oxygen-induced retinopathic model, by stimulating production of angiopoietin-2 and thereby driving angiogenesis (17).

In this study, we tried to identify an association of HIF1α with the redox system, Krebs cycle, ubiquitin proteasomal complex, and other regulatory proteins. We will also explore the relationship of HIF1α with notch1 to determine whether notch1 has a tumor-suppressive or oncogenic role in CML so that it can be used for the diagnosis of CML. Despite the success of TKIs, the search for new therapeutic options is still important due to the emergence of primary or secondary resistance to treatment in patients and the difficulty of eradicating CML stem cells as the main “culprit” of the disease.

## Material and Methods

### Patient’s Samples

Human CML samples (n = 60; blood: 60, bone marrow aspirates: 10) and healthy control samples (n = 20) were obtained from the Department of Pathology and Biochemistry at King George’s Medical University after informed consent was obtained from the patients. The clinical diagnosis of CML was based on the patient’s presentation, morphology on a peripheral blood smear, and bone marrow by using Leishman staining. Further immunophenotyping of blast cells was performed by flow cytometry by taking markers such as CD34, CD33, CD14, CD20, CD10, CD19, HLA-DR, TdT, CD2, CD3, CD5, CD7, CD13, CD19, CD20, CD23, CD45, CD64, CD79a, CD117, and CD 200. Patient characteristics are presented in **Table 1**. Most of the experiments were performed immediately after taking samples in the Department of Biochemistry. Aliquots of samples (whole blood and bone marrow tissue) were frozen in complete RPMI 1640 media (Gibco) and subsequently stored in liquid nitrogen. Whenever needed for any experiment, cells were thawed and suspended in prewarmed RPMI 1640 with 40% FBS at 37°C. Cells were washed and allowed to recover for 45 minutes in the same medium at 37°C. Cells were washed again and suspended in PBS with 0.1% BSA.

**Table 1:** Overview of clinical characteristics of CML patients.

S.No.	Patients Characteristics	
1.	No. Of Patients	60
2.	Healthy Control	20
3.	Male/Female	36/24
4.	Specimen	Peripheral blood/Bone marrow (60/10)
5.	Age at diagnosis, year median (range)	49.7 (16-91)
6.	Blast cells range	86% (Average)
7.	Markers combinations (Bright)	CD2, CD5, CD7, CD10, CD13, CD14, CD19, CD20, CD23, CD45, CD64, CD79a, CD117, CD200 etc.

### Peripheral Blood Smear, and Bone Marrow Staining

Leishman dye was prepared in methanol (0.2 g of Leishman powder and dissolved in 100 mL of methanol). After preparing blood films, it is allowed to air dry. The slides were flooded with Leishman stain for 2 minutes and washed in a stream of buffered water for 2 minutes to acquire a pinkish tinge. In cases of suspicion of leukemia, blood films made from buffy coat preparation were stained with Leishman’s stain (47).

### Blast Cells Conformation by Flow Cytometry

Monoclonal antibody combinations consisted of fluorescein isothiocyanate (FITC)-, phycoerythrin (PE)-, peridinyl chlorophyllin (PerCP)-, and phycoerythrin-Cy7 (PE-Cy7)-labeled monoclonal antibodies. Anti-CD45 V500-A, anti-CD34 PercP-A, anti-CD33 APC-A, anti-CD79a, APC-A, anti-CD13-PE-A, anti-CD19-PE, anti- CD25-FITC-A, anti-CD20-V450-A, anti-CD7-FITC-A, anti-CD10-PE, anti- CD19-PE, anti-CD20-V450-A, anti-CD14-APC, anti-CD-64 FITC-A, anti-CD117-PE, human leukocyte antigen (HLA)-DR-APC, anti-CD14-APC, and many more antibodies were purchased from BD Biosciences (San Jose, CA, USA). We followed the stain lyse wash method; the appropriate number of FACS tubes was labeled for the name of the patient and the combination of fluorochrome-conjugated monoclonal antibodies. In each tube, 100 μL of the sample (bone marrow aspirate or whole blood) was pipetted into the tube and poured with 20 μL of antibody/antibody cocktail in the respective tubes, which was incubated in the dark for 10-15 minutes. After incubation, 2 mL of diluted FACS lyse solution was added to each tube. The samples were then centrifuged at 200-300 g for 3-5 minutes. The supernatant was discarded, the pellet formed at the bottom of the tube was broken, and the remaining cells were washed twice with shealth fluid. The cells were again resuspended in approximately 0.5 mL of shealth fluid and run on precalibrated flow cytometry. Data acquisition was performed by using FACSCanto (BD Biosciences).

### BCR-ABL Translocation by Multiplex PCR

A multiplex RT-PCR assay was performed with a Seeplex leukemia BCR/ABL kit (Seegene, Seoul, Korea), which was predesigned to detect eight common types of BCR/ABL transcripts, including micro, minor, and major breakpoint cluster regions (M-bcr, m-bcr, and μ-bcr), and followed the manufacturer’s instructions. The cycling conditions were as follows: 94°C for 15 minutes (1 cycle); 94°C for 30 seconds, 60°C for 1 minute 30 seconds, 72°C for 1 minute 30 seconds (37 cycles); and 72°C for 10 minutes (1 cycle). The PCR products were analyzed with 2% agarose gel electrophoresis at 100 V for 60 minutes.

### Redox Assay

All oxidative and antioxidant protocols followed as explained in previous literature along with their customization on 96-well plates, ferrous ion level (48), ROS detection by DCFDA (49), glutathione reductase (50), catalase (51), superoxide dismutase (52), lipid peroxidation (53), and protein carbonyl (54). ROS/SOD by Abcam-ab139476 kit, Nitric oxide assay kit (colorimetric)- ab65328, protein carbonyl content assay kit- ab126287, and Total Antioxidant assay kit- Sigma Aldrich; CS0790. All protocols were performed according to the manufacturer’s instructions.

### DNA Fragmentation Assay

DNA fragmentation (TaKaRa 6137) was detected by agarose gel electrophoresis and comet assay according to the Abcam protocol (ab238544).

### Kreb’s Cycle Metabolites Measurement

Fumarate, malate, and succinate quantification from serum samples was performed using a commercially available kit (Abcam ab102516, ab83391, and ab204718, respectively) following the manufacturer’s instructions.

### Gene Expression

Total RNA from cells was isolated by following the TRIzol method. The concentration of RNA and their structural integrity were confirmed by using a Nanodrop 2000 UV–Vis spectrophotometer (Thermo Scientific). Only RNA with the ratios from 1.9- 2.0 of absorbance at 260/280 nm has been used. The isolated mRNA was reverse-transcribed using the High-Capacity cDNA Reverse Transcription Kit (4368814) according to the protocol. Quantitative reverse transcription-PCR (q RT-PCR) was performed by using PowerUp SYBR green master mix (ABI- A25741) on a 7500 Fast Real-Time PCR system (Applied Biosystem, Thermo Scientific). Quantification was performed with the ΔΔ Ct method with β-actin serving as a reference gene, and the RT-PCR results were analyzed by DataAssist software (Thermo Scientific). The oligonucleotide primers are listed in **Table S1**. All primers were analyzed with positive controls by performing melting profiles following q RT-PCR, and product sizes were checked by 2.2% agarose gel electrophoresis. PCR conditions were as follows: 40 cycles of 15 seconds at 95°C, 15 seconds at annealing temperature (60°C for all other genes), and 15 seconds at 72°C. Specimens were assayed in duplicate for at least three independent experiments as indicated.

### Western Blotting

Blood and bone marrow were harvested, and the proteins were isolated by lysis buffer (RIPA Buffer) and measured using the BCA protein assay method with a spectrophotometer (Thermo Scientific) at 562 nm. Protein samples were separated with 15% SDS-polyacrylamide gel electrophoresis (SDS-PAGE) and transferred onto PVDF membranes (Bio-Rad). Immune complexes were formed by incubation of the proteins with primary antibody overnight at 4°C. Blots were washed and incubated for 1 h with hrp-conjugated secondary antibodies. Immunoreactive protein bands were detected with an Odyssey Scanning System (LI-COR Inc., Lincoln, NE, USA).

### LCMS/MS Protein Profiling

For LC-MS/MS analysis, whole protein was isolated through RIPA buffers, and 2 sets of samples were taken for consideration (CML pooling with 10 samples and control pooling with 10 samples). The quality of the samples was checked by running 1D gel electrophoresis (SDS-PAGE), and protein was estimated spectrophotometrically by the BCA method.

### In Solution Protein Digestion Before LC‐MS/MS

100μg of the protein sample was taken for digestion. After digestion, the sample was diluted with 50 mM NH4HCO3, and then the protein sample was treated with 100 mM DTT at 95°C for 1 hr followed by 250 mM IDA (iodoacetamide) at room temperature in the dark for 45 minutes and digested with trypsin, which was incubated overnight at 37°C. The resulting sample was vacuum dried and dissolved in 50 μl of 0.1% formic acid in water. After centrifugation at 10000 g, the supernatant was collected into a separate tube.

### Protein Identification by LC‐MS/MS

Ten microliters of the cleaned sample was injected onto a BHE C18 UPLC column for separation of peptides (**Supplementary Table**), followed by analysis on a Waters Synapt G2 Q-TOF instrument for MS and MS/MS with an ESI source. The raw data were analyzed to obtain the complete integrated sequence of the sample by MassLynx 4.1 WATERS, peptide editor software. The individual peptide MSMS spectra were matched to the database sequence with the help of PLGS software, WATERS. The instrument used for acquiring mass spectrometry data was UPLC connected with Waters Synapt G2 (QTOF). The parameters used for identification are already mentioned, such as peptide mass tolerance at the MS1 level of 50 ppm and fragment mass tolerance at the MS2 level of 100 ppm. During the processing of the sample, cysteine sites were modified to carbamidomethylated cysteine, and the methionine sites were prone to oxidation, which was considered a variable modification to the mass (55–57). Quantitive details are given in **Table S2**.

### Protein-Protein Interaction (PPI) Network Analysis

The search tool for retrieval of interacting genes (STRING) (https://string-db.org) database, which integrates both known and predicted PPIs, can be applied to predict functional interactions of proteins. To seek potential interactions between all the proteins included in this study, the STRING tool was employed, corresponding to CML cells. Active interaction experimental proteins, which were limited to humans only, and an interaction score > 0.4 were applied to construct the PPI networks. In the networks, the nodes correspond to the proteins, and the edges represent the interactions.

### Statistical Analysis

All analyses were performed by using GraphPad Prism-9, SPSS 16.0 version, from which we performed Student’s t-test, one-way ANOVA, Chi-square test, Pearson correlation, and ROC curve analysis (Metaboanalyst version 5.0). All comparisons were made relative to untreated controls, and significance of the difference is indicated as *p<0.01, **p< 0.001, ***p< 0.0001, ****p<0.00001. All quantitative data presented are the mean ± SD from at least three samples per data point.

## Result

### Clinical Diagnosis of Human Chronic Myeloid Leukemia (CML)

**Figure 1:**
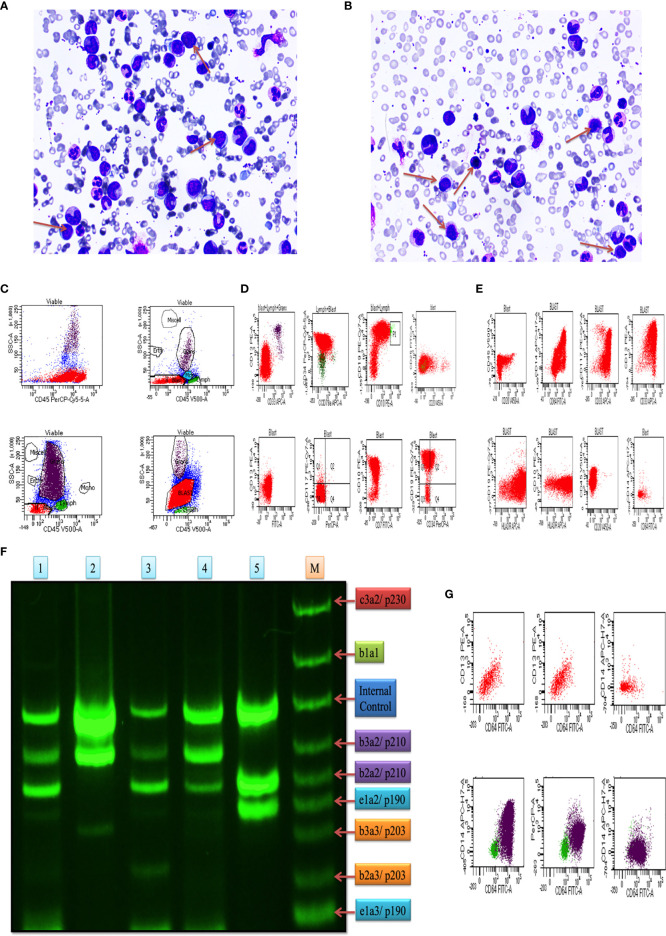
Clinical diagnosis was confirmed on bone marrow aspiration, marrow trephine biopsy, and BCR/ABL1 translocation. **(A, B)** Representative images of Leishman staining of the blood of CML patients, where blast crisis cells are shown by the red arrow. **(C)** Flow cytometry results show the blast cells in various patients of CML patients, which were plotted on CD45 with side scatter and plotted on the marker and marker vs marker for the confirmation of CML (see also **Figure S1**). **(D)** the first two rows of the scattered plot show the myeloid blast crisis cells, and other two rows show lymphoid blast crisis cells. **(E)** the scattered plot shows the blast crisis phase of chronic myeloid leukemia. **(F)** shows BCR/ABL1 translocation in CML patients, where lanes 1-5 show p210/p190 translocation, and lane M shows the standard of all kinds of translocation. **(G)** CD64 marker plot showing that it is present in all kinds of cells in CML patients and responsible for the production of ROS intermediates.

In this study, we included blood and bone marrow tissues of CML patients. The morphology of blast crisis cells from peripheral blood smears, bone marrow aspirate smears, and bone marrow trephine biopsy specimens, along with immunophenotypic findings and flow cytometry, is shown in **Figures 1A, B**), which shows myeloid blast crisis cells stained by Leishman dye. Furthermore, flow cytometry results help identify phenotypes with different biomarkers, such as CD34, CD33, CD14, CD20, CD10, CD19, HLA-DR, TdT, CD2, CD3, CD5, CD7, CD13, CD19, CD20, CD23, CD45, CD64, CD79a, CD117, and CD 200. Blast cells were gated on CD45 vs side scatter. The expression of myeloid markers (CD13, CD33, CD117, CD14, CD64, cMPO), B-lymphoid markers (CD19, CD20, CD79a, CD10), T-lymphoid markers (CD3, cytoplasmic CD3, CD2, CD5, CD7) and immaturity markers (CD34, TdT, HLA-DR) was studied. We performed flow cytometry analysis for different patients, as shown in **Figures 1C–E**. The results are summarized in **Table 1** and extension in **Table S3**. Ninety percent of blast cells gated on dim CD45 and extended to the monocytic region on CD45 versus the side scatter plot, as shown in **Figure 1G**. A reliable number of CD45 events (20,000) were measured, and we found that 95% of patients had approximately 15,000 blast cells in 20000 events. Flow cytometry data confirmed the presence of blast cells in CML patients.

### BCR/ABL1 Translocation in Blast Crisis Cell of CML

Plot results of the multiplex RT-PCR lane showed maximum p210BCR/ABL1 translocation in the blast phase. After the analysis by ImageJ, we found expression coverage in lane 1; b2a2/p210: 14.7%, b2a2/p210: 27.4%, in lane 2; b3a2/p210: 64.1%, in lane 3; b3a2/p210: 8.5%, b2a2/p210: 18.4%, in lane 4; b3a2/p210: 30.2%, b2a2/p210; 19.2%, in lane 5; b3a2/p210: 60.2%, b2a2/p210: 27.5%. Lane M shows the standard BCR/ABL1 translocation for comparison. Overall, the results indicated that p210/p190 translocation was maximum in all blast crisis cells of CML patients, as shown in **Figure 1F**.

### Role of the Redox System in the Pathogenesis of CML

**Figure 2:**
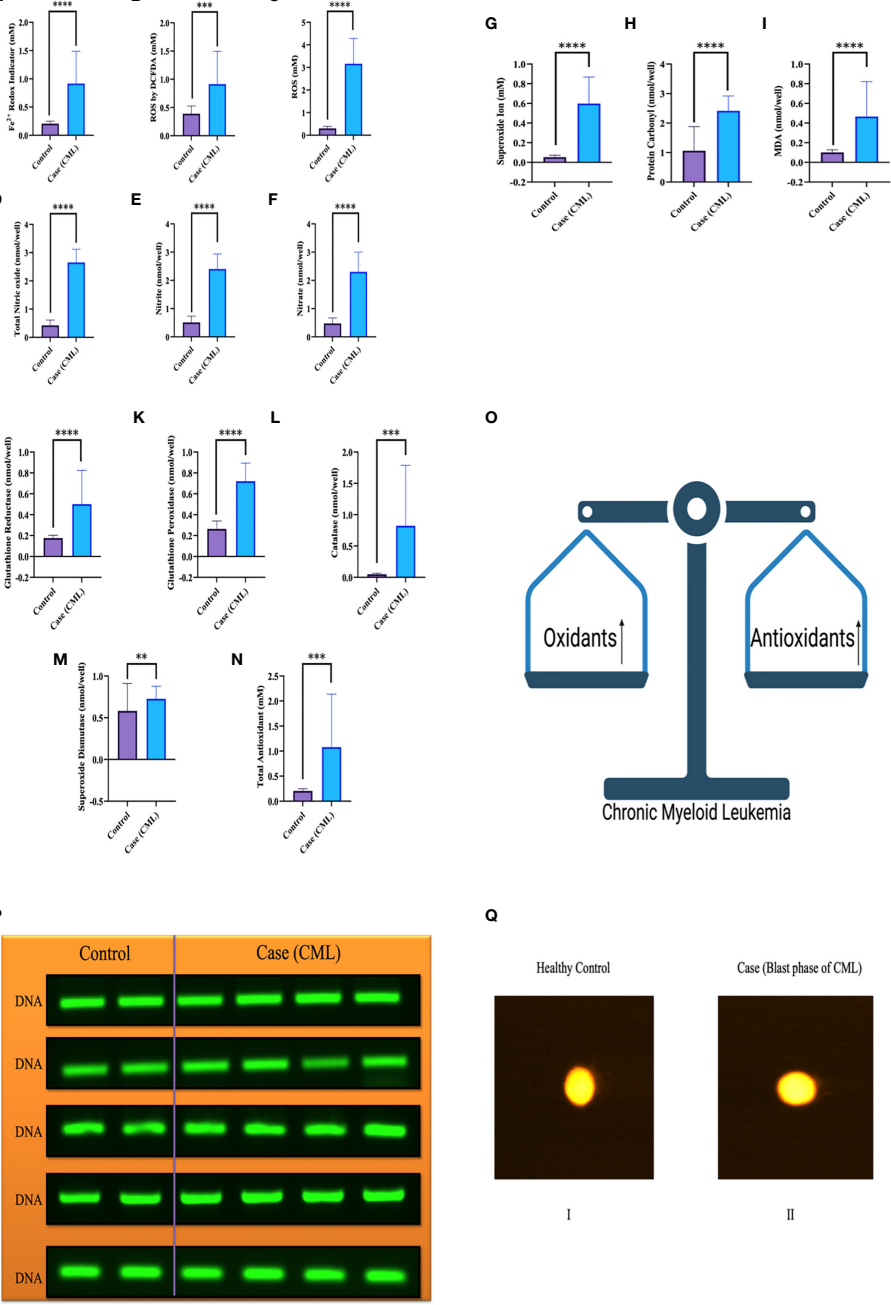
Redox system in the pathogenesis of CML and DNA fragmentation. **(A–I)** Significant oxidative parameters and their overexpression were found in CML patients. **(J–O)** Antioxidant levels were also found to be high in CML due to their pathogenesis and protection from apoptosis. Both oxidative and antioxidative factors were found to be high and make an equilibrium for survival. **(P, Q)** shows that the integrity of DNA was maintained when both parameters were upregulated, which triggered morphological changes in CML cells but was not able to damage DNA integrity. All the data are the mean ± SD, ****p<0.00001, ***p<0.0001, **p<0.001; Student’s t test (paired).

We collected all samples (plasma, serum, whole blood lysates and bone marrow tissues) to better elucidate the whole-body response. Here, we found that oxidative parameter levels were significantly higher in blast crisis cells than in healthy controls. Fe^2+^ level in case 0.76 ± 0.48 mM, control 0.1984 ± 0.04 mM with p = 0.003, ROS level by DCFDA in case 0.6680 ± 0.5 mM, control 0.4269 ± 0.12 mM with p = 0.1, ROS level in case 3.184 ± 1.15 mM, control 0.3074 ± 0.091 mM with p <0.0001, total nitric oxide level in case 2.643 ± 0.4 nmol/well, control 0.4135 ± 0.18 nmol/well with p <0.0001, nitrite level in case 2.372 ± 0.5 nmol/well, control 0.5024 ± 0.2 nmol/well with p <0.0001, nitrate level in case 2.3 ± 0.6 nmol/well, control 0.4746 ± 0.2 nmol/well with p <0.0001, superoxide ion concentration in case 0.5013 ± 0.2 nmol/well, control 0.05 ± 0.01 nmol/well with p <0.0001, MDA level in case 0.14 ± 0.02 nmol/well, control 0.1 ± 0.01 nmol/well with p <0.0001, and protein carbonyl (PC) in case 2.25 ± 0.5 nmol/well, control 1.47 ± 0.7 nmol/well with p = 0.0004 as ahown in **Figure 2A–I**. On the other hand, antioxidant parameters were also found to be significantly higher in blast crisis cells of CML. Glutathione reductase in case 0.26 ± 0.08 nmol/well, control 0.17 ± 0.02 nmol/well with p = 0.0002, glutathione peroxidase in case 0.67 ± 0.3 nmol/well, control 0.26 ± 0.07 nmol/well with p <0.0001, catalase level in case 0.31 ± 0.24 nmol/well, control 0.05 ± 0.01 nmol/well with p <0.0001, superoxide dismutase (SOD) expression in case 0.74 ± 0.052 nmol/well, control 0.61 ± 0.05 nmol/well with p = 0.01, and total antioxidant concentration in case 1.164 ± 0.16 mM, control 0.2074 ± 0.044 mM with p = 0.001 as shown in **Figures 2J–N**. Oxidants and antioxidants were found to be high in blast crisis cells of CML (**Figure 2O**), which helped in pathogenesis without damaging the DNA, as confirmed by agarose electrophoresis examination and comet assay, as shown in **Figures 2P, Q**. Overall, the results indicated that oxidative stress only changed the morphology of cells through lipid peroxidation and protein carbonyl generation, but DNA fragmentation was significantly prevented by antioxidant factors. All the quantitative data are presented in **Table 2** with extended information in **Table S6**.

**Table 2:** Quantitative details of oxidative and antioxidative parameters.

S.No.	Factors	Control (Mean ± SD)	Case (Mean ± SD)	*p* value
1.	Fe ^2+^ level (mM) Redox Indicator	0.2 ± 0.04	0.9 ± 0.5	<0.0001
2.	ROS (mM) by DCFDA	0.39 ± 0.13	0.9 ± 0.5	0.0002
3.	ROS (mM)	0.3 ± 0.09	3.1 ± 1.1	<0.0001
4.	Glutathione Reductase (nmol/well)	0.17 ± 0.02	0.5 ± 0.32	<0.0001
5.	Glutathione Peroxidase (nmol/well)	0.2 ± 0.07	0.7 ± 0.17	<0.0001
6.	Catalase (nmol/well)	0.04 ± 0.01	0.82 ± 0.96	0.0008
7.	SOD (nmol/well)	0.5 ± 0.3	0.74 ± 0.15	0.0095
8.	MDA (nmol/well)	0.1 ± 0.01	0.4 ± 0.3	<0.0001
9.	Protein Carbonyl (nmol/well)	1.06 ± 0.8	2.4 ± 0.5	<0.0001
10.	Total Nitric Oxide (nmol/well)	0.42 ± 0.18	2.6 ± 0.4	<0.0001
11.	Nitrite (nmol/well)	0.51 ± 0.2	2.4 ± 0.5	<0.0001
12.	Nitrate (nmol/well)	0.4 ± 0.2	2.3 ± 0.6	<0.0001
13.	Total Antioxidant (mM)	0.2 ± 0.04	1.08 ± 1.05	0.0006
14.	Superoxide ion (nmol/well)	0.05 ± 0.01	0.59 ± 0.2	<0.0001

### Redox Factors Increase Krebs Metabolites, Which Are Involved in the Oncogenesis of CML

**Figure 3:**
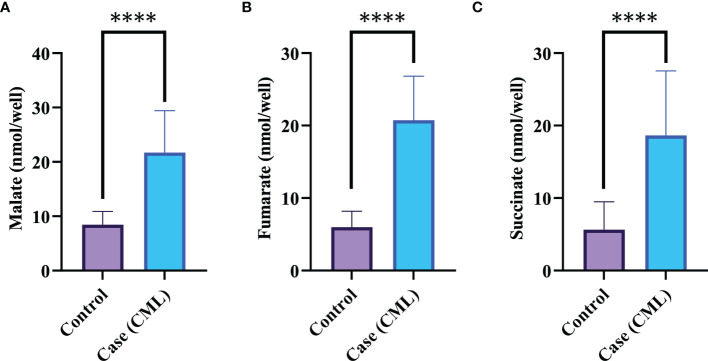
Krebs metabolite in CML progression. **(A–C)** Krebs cycle oncometabolites in CML were quantified by colorimetry. Malate, fumarate, and succinate levels were found to be high in CML patients, with fold changes of 1.3609, 1.709, and 1.7231, respectively. All data are mean ± SD, ****p<0.00001, Student’s t-test (paired).

Mitochondria are cellular organelles that generate ATP and metabolites for survival and growth, respectively. Abnormal accumulation of succinate and fumarate leads to inhibition of PHDs, resulting in HIF1α stabilization. We studied three oncometabolites (malate, succinate, and fumarate) in blood lysate, serum, plasma, and bone marrow tissues of blast crisis cells of CML patients. However, all three oncometabolites were significantly upregulated in the patients (20.2 ± 6.71 nmol/well, 13.46 ± 7.56 nmol/well, and 18.55 ± 4.09 nmol/well) compared with healthy controls (8.09 ± 2.46 nmol/well and 5.03 ± 3.85 nmol/well), with p <0.0001, 0.001, and <0.0001 for malate, succinate, and fumarate, respectively, as shown in **Figures 3A–C** and **Table 3** (also see **Table S6**).

**Table 3:** All the quantitative details of Krebs cycle oncometabolite.

S.No.	Factors	Control (Mean ± SD)	Case (Mean ± SD)	*p* value
1.	Malate (nmol/well)	8.4 ± 2.4	21.7 ± 7.6	<0.0001
2.	Succinate (nmol/well)	5.6 ± 3.8	18.6 ± 8.9	<0.0001
3.	Fumarate (nmol/well)	5.9 ± 2.19	20.7 ± 6.08	<0.0001

### Redox and Krebs Oncometabolites Might Activate and Stabilize Hypoxia-Inducible Factor (HIF1α), Which Alters Other Regulatory Proteins

**Figure 4:**
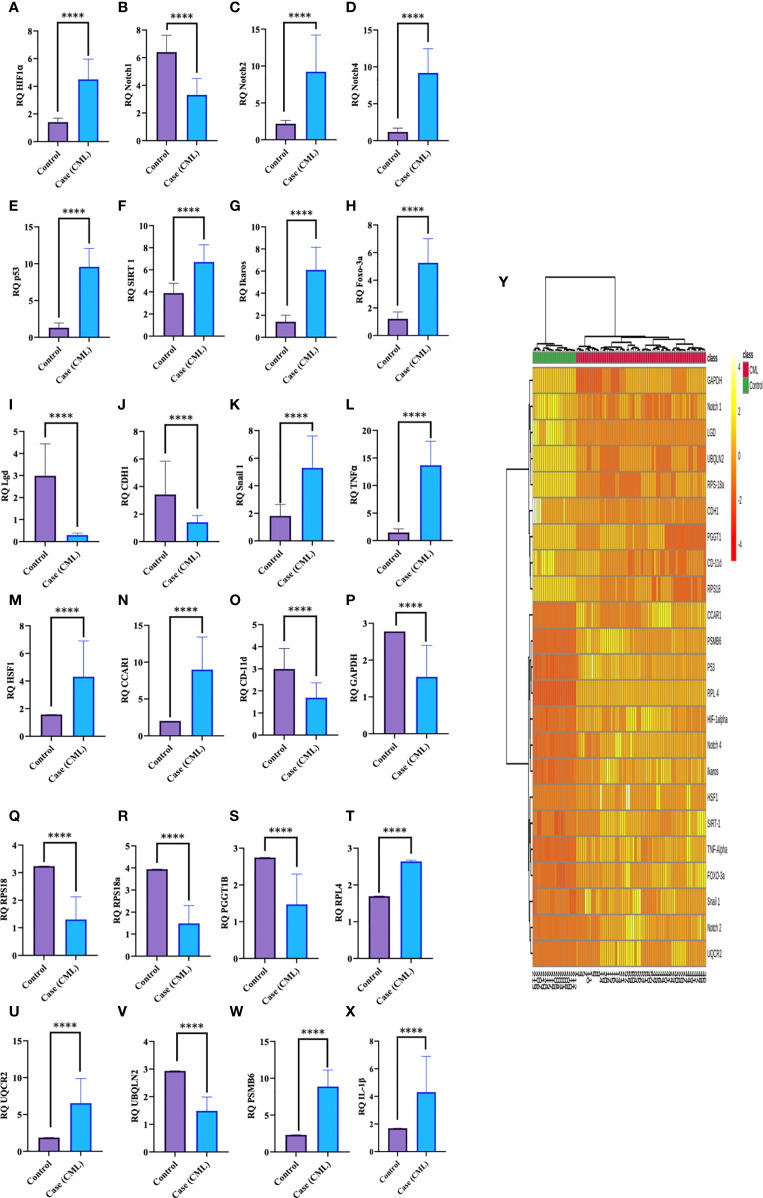
Gene expression of HIF1α, Notch1, and their associated genes in CML. **(A–X)** Gene expression was quantified by RT-PCR in CML (n = 60) and healthy controls (n = 20), where HIF1α and its associated genes were found to be upregulated and Notch 1 was downregulated in blast crisis cells of CML patients. **(Y)** The heat map shows the relative expression of all the genes in CML (class indicated Control and CML), and the intensity of color shows the expression of genes. All quantitative data are the mean ± SD, ****p<0.00001, Student’s t-test (paired).

**Figure 5:**
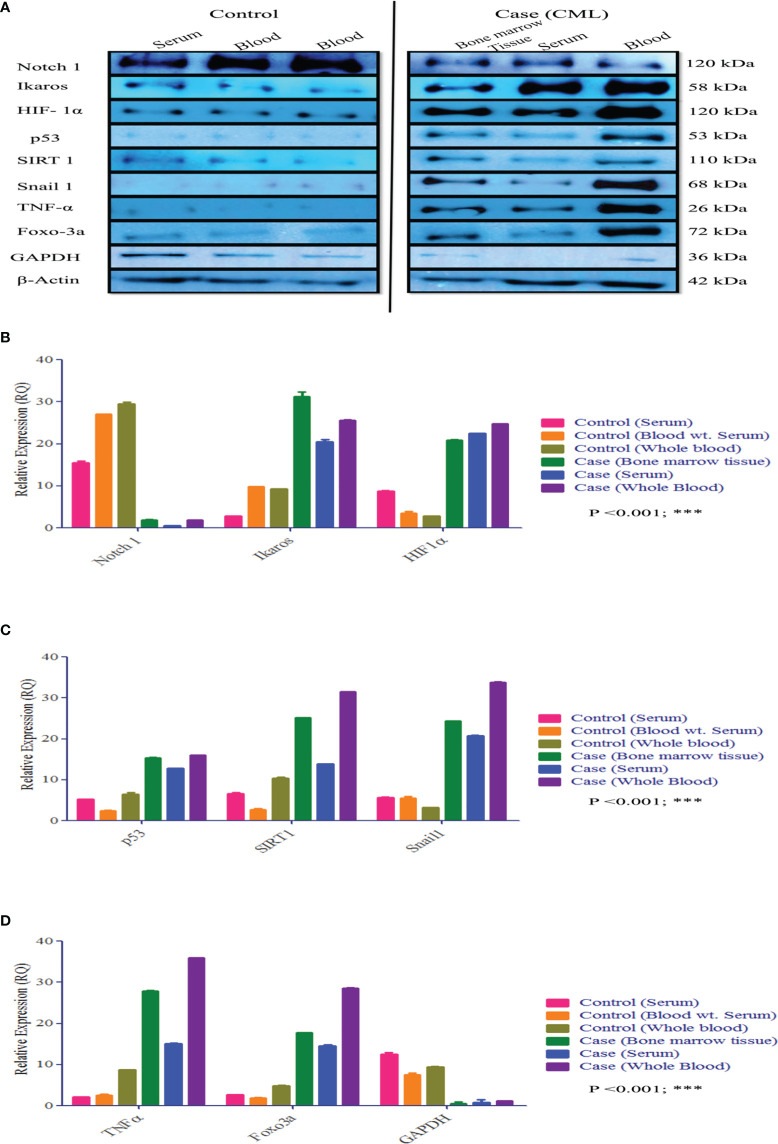
Protein expression of HIF1α, Notch1, and their associated genes in CML. **(A–D)** Western blot and densitometric analysis of the proteins. In healthy controls, we performed Western blotting in blood and serum, wherein CML was performed Western blotting in bone marrow tissue, serum, and blood and compared all the results with healthy controls. All quantitative data are mean ± SD, ***p<0.001, ANOVA.

Redox and Krebs oncometabolites might stabilize HIF1α, which was further confirmed at the mRNA and protein levels. In this section, we also included genes that directly or indirectly participate in the pathogenesis of CML. The genes taken together for the study were divided into two parts: oncogenic and tumor suppressive. The oncogenic genes that were found to be significantly (p <0.0001) highly expressed in blast crisis cells included HIF1α (fold change: 1.8007), Notch 2 (fold change: 2.092), Notch 4 (fold change: 2.9638), Ikaros (fold change: 2.1033), Snail1 (fold change: 1.5436), p53 (fold change: 2.8633), TNFα (fold change: 3.2131), CCAR1 (fold change: 2.1591), SIRT1 (fold change: 0.78546), Foxo-3a (fold change: 2.1207), HSF1 (fold change: 1.4555), UQCR2 (fold change: 1.8107), PSMB6 (fold change: 1.9391), RPL4 (fold change: 0.6395), and IL-1β. On the other hand, genes that are found to be tumor suppressive in the blast crisis cells of CML patients are Notch1 (fold change: -0.95244), CDH1 (fold change: -1.2873), Lgd (fold change: -3.3169), CD11d (fold change: -0.82285), UBQLN2 (fold change: -0.97842), RPS18/18a (fold change: -1.3097/-1.4092), PGGT1B (fold change: -0.89755), and GAPDH (fold change: -0.84476), which are significantly (p <0.0001) downregulated, and their fold change is mentioned in **Table 4** (see also **Table S6**) and **Figures 4A–Y**. Therefore, GAPDH did not behave like an internal control in this study, as it was altered in CML cells. Next, we validated the expression level by western blot, where we found that Notch1 and GAPDH were downregulated in CML, while Ikaros, HIF1α, p53, SIRT1, TNFα, and Foxo-3a were upregulated in CML (β-actin as an internal control), as shown in **Figures 4A–D**. When comparing western blot results, we found that the expression of all genes was significantly higher, but most of them were expressed in whole blood compared with serum and bone marrow tissue as shown in **Figures 5A–D**. From this outcome, we can infer that whole blood might be best for the diagnosis of CML at the molecular level. Both the RT-PCR and western blot results indicate that HIF1α is oncogenic and upregulated when the Redox and Krebs oncometabolites are also highly expressed in CML; on the other hand, Notch1 behaves like a tumor suppressor in CML cases.

**Table 4:** Relative expression of genes by RT-PCR.

S.No.	Gene	RQ Control (Mean ± SD)	RQ Case (Mean ± SD)	P-value
1.	Beta-actin	1	1	–
2.	Notch 1	6.4 ± 1.2	3.3 ± 1.1	<0.0001
3.	Notch 2	2.1 ± 0.4	9.2 ± 4.9	<0.0001
4.	Notch 4	1.1 ± 0.5	9.1 ± 3.2	<0.0001
5.	Ikaros	1.4 ± 0.5	6.1 ± 2.04	<0.0001
6.	CDH1	3.4 ± 2.4	1.4 ± 0.4	<0.0001
7.	Snail1	1.8 ± 0.8	5.2 ± 2.3	<0.0001
8.	P53	1.3 ± 0.6	9.5 ± 2.4	<0.0001
9.	CD11d	2.9 ± 0.9	1.6 ± 0.6	<0.0001
10.	TNF-Alpha	1.4 ± 0.6	13.6 ± 4.3	<0.0001
11.	CCAR1	2.01 ± 0.001	8.9 ± 4.4	<0.0001
12.	SIRT1	3.8 ± 0.8	6.7 ± 1.5	<0.0001
13.	FOXO-3a	1.2 ± 0.4	5.2 ± 1.7	<0.0001
14.	HIF-1alpha	1.4 ± 0.2	4.5 ± 1.4	<0.0001
15.	HSF1	1.5 ± 0.03	4.3 ± 2.5	<0.0001
16.	Lgd	2.9 ± 1.4	0.29 ± 0.08	<0.0001
17.	UQCR2	1.8 ± 0.01	6.5 ± 3.3	<0.0001
18.	UBQLN2	2.9 ± 0.002	1.4 ± 0.4	<0.0001
19.	PSMB6	2.3 ± 0.002	8.8 ± 2.2	<0.0001
20.	RPS18	3.2 ± 0.003	1.3 ± 0.8	<0.0001
21.	RPS18-A	3.9 ± 0.002	1.4 ± 0.8	<0.0001
22.	PGGT1B	2.7 ± 0.002	1.4 ± 0.8	<0.0001
23.	RPL4	1.6 ± 0.002	2.6 ± 0.035	<0.0001
24.	GAPDH	2.7 ± 0.0003	1.5 ± 0.8	<0.0001

### LC/MS Study Explored the Regulatory Role of HIF1α on Notch1, Ubiquitin Proteasomal Proteins, Apoptotic Proteins, and Some Other Regulatory Proteins

**Figure 6:**
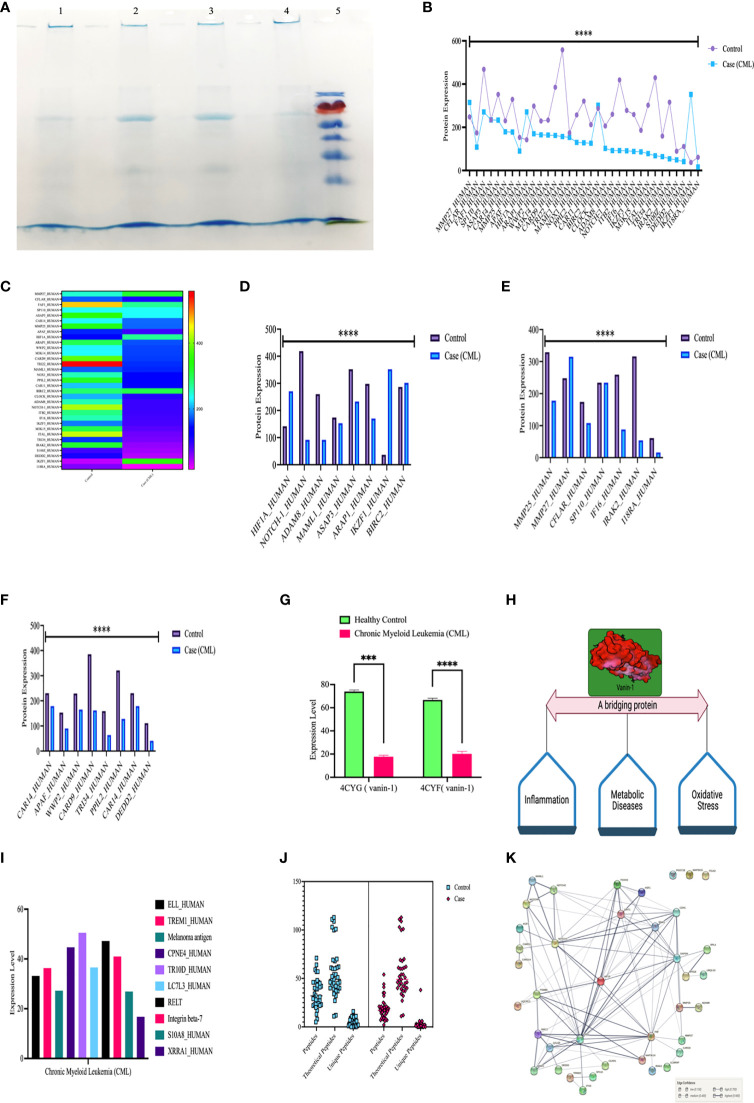
High-throughput screening of HIF1α, Notch1, and all the proteins in CML by LC/MSMS. **(A)** SDS-PAGE shows the integrity of the protein, lanes 1 and 2 show the healthy control, lanes 3 and 4 show the CML, and lane M shows the protein ladder. **(B, C)** Shows the 37 protein sets that are relative compared in both groups through a heatmap. **(D–F)** Comparison of HIF1α, Notch1, and their associated proteins, where the graph shows that apoptotic and ubiquitin proteasomal protein sets are downregulated under CML conditions. **(G, H)** Vanin 1 expression was negligible in CML cases, which are connecting links between inflammation and oxidative stress, and their equilibrium was disrupted in blast crisis cells. **(I)** The graph shows the proteins that were only expressed in CML cases. **(J)** Estimation plot shows that the peptides of the abovementioned proteins were significantly lower under CML conditions. **(K)** Protein-protein interaction of all the proteins involved in this study and divided into the different domains where HIF1α acts as a master regulator of all the genes. All quantitative data are the mean ± SD, ****p<0.0001, ***p<0.001, ANOVA and chi-square test.

We further validated our results by high-throughput screening by LC/MS at the protein level (protein array). We isolated whole protein from bone marrow tissues and pooled them and then further performed LC/MS, where we found expression of approximately 800 proteins in control and 700 in the case and few peptides. For this study, we included 39 proteins, which were comparable in both the case and control, showing their relative expression by heat map, as shown in the figure. Approximately 10 proteins were present specifically in CML cases and were only responsible for the progression of leukemia. Based on protein-protein interactions, we categorized 49 protein sets into five different domains: the first domain included HIF1α, notch1/cleaved proteins, Ikaros, and survivin; the second domain consisted of interferon factors; the third domain consisted of apoptotic and ubiquitination factors; and the fourth domain included Vanin1. First domain proteins ASAP3 (ankyrin repeat and PH domain-containing protein 3), ARAP1 (Arf-GAP with Rho-GAP domain), MAML1, ADAM8, Notch1 were found to be significantly down expressed in CML cases, while HIF1α, IKZF1 (Ikaros), BIRC2 (inhibitor of apoptosis protein 2/Survivin) were upregulated in case. The second domain shows that MMP27, CFLAR (FADD-like apoptosis factor), S110 (interferon-induced protein), MMP25, IFI16 (gamma interferon-inducible protein 16), IRAK2 (interleukin 1 receptor-associated kinase-like 2), and 18RA (interleukin 18 receptor accessory protein) were significantly downregulated in blast crisis cells of CML patients. The third domain indicates that CAR14 (Caspase recruitment domain-containing protein 14), APAF (apoptotic protease activating factor 1), WWP (NEED4-like E3 ubiquitin-protein ligase), M3K14 (Mitogen activating protein kinase 1), CARD9 (Caspase recruitment domain-containing protein 9), TRI22 (E3 ubiquitin-protein ligase), PPIL2 (Ring type E3 ubiquitin-protein ligase), CAR11 (Caspase recruitment domain-containing protein 11), M3K15 (Mitogen-activated protein kinase 15), DEDD2 (DNA binding death effector domain-containing protein 3) were found to be significantly downregulated in CML patients. The fourth domain protein, Vanin-1, which is a unique protein that balances inflammation, metabolic diseases, and oxidative stress, was highly expressed in the control group but showed negligible expression in CML (p <0.0001). CML-specific proteins (ELL; eleven nineteen lysine-rich leukemia protein, TREM1; triggering receptor expressed on myeloid cells, LC7L3; cisplatin resistance overexpressed protein) were found to be upregulated in blast crisis cells, as shown in the **Figures 6A–J** and quantiative details presented in **Table S4**, and unique peptides were also found to be low in CML cases. The protein-protein interaction (PPI) shows a strong interaction with all domains, and their master regulator is HIF1α, as shown in **Figure 6K**. We included more than 0.9 edge scores, as presented in **Supplementary Table S5**.

### The Pearson Correlation of the HIF1α Gene With All the Factors

**Figure 7:**
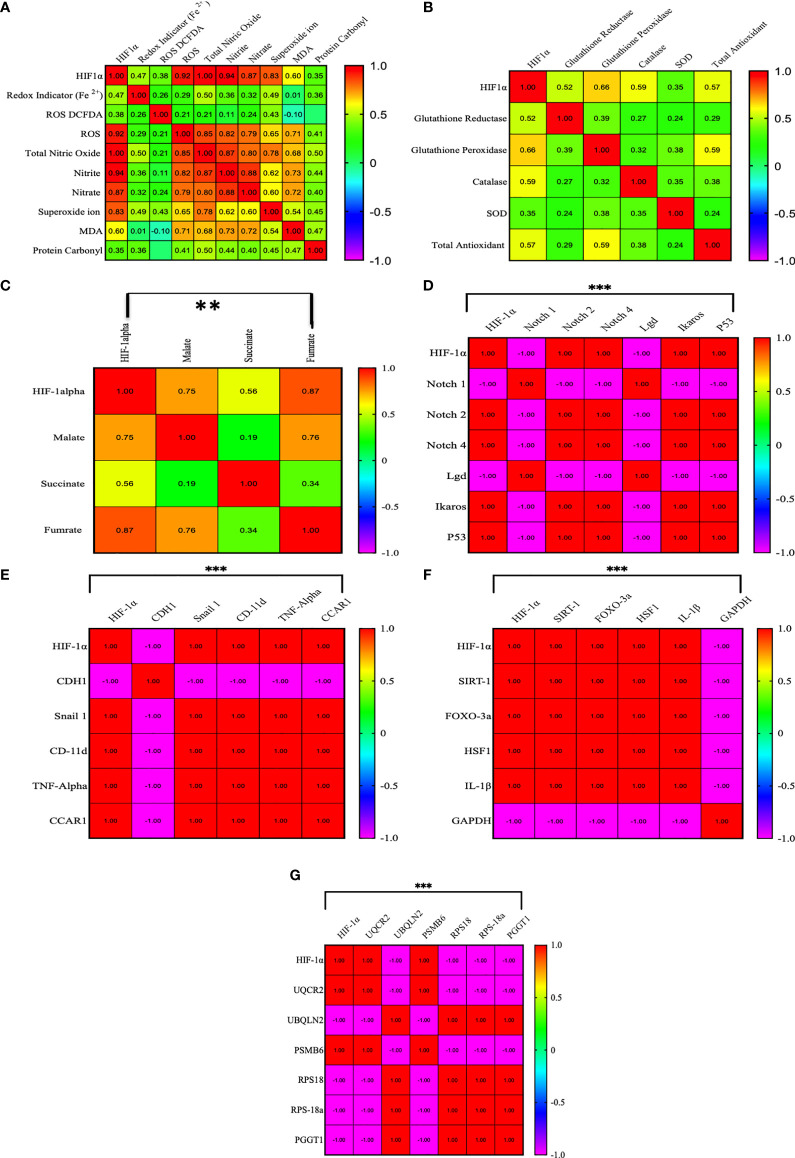
Pearson correlation between HIF1α and all the factors. **(A–G)** HIF1α has a strong positive correlation with ROS (0.922, p = 0.01), total NO (0.99, p = 0.01), nitrite (0.94, p = 0.01), nitrate (0.873, p = 0.01), and superoxide ions (0.827, p = 0.01), while HIF1α does not show a strong correlation with other redox factors. However, HIF1α had a positive correlation with malate (0.751, p = 0.01) and fumarate (0.871, p = 0.01) and a moderate positive correlation with succinate (0.562, p = 0.01). HIF1α also showed a strong positive correlation with Notch 2, Notch 4, Ikaros, p53, Snail 1, CD-11d, TNFα, CCAR1, SIRT1, Foxo-3a, HSF1, IL-1β, UQCR2, and PSMB6 (1.00, p = 0.001) and a strong negative correlation with Notch1, Lgd, CDH1, GAPDH, UBQLN2, RPS18/18a, and PGGT1B (-1.00, p = 0.001). **p >0.001; ***p >0.0001.

We established a correlation of HIF1α with redox parameters, Krebs oncometabolites, and associated genes. In the correlation matrix, the intensity of color indicates the strength of correlations, red color shows a strong positive correlation, and blue color shows a strong negative correlation. HIF1α has a strong positive correlation with ROS (0.922, p = 0.01), total NO (0.99, p = 0.01), nitrite (0.94, p = 0.01), nitrate (0.873, p = 0.01), and superoxide ions (0.827, p = 0.01), while HIF1α does not show a strong correlation with other redox factors. However, HIF1α had a positive correlation with malate (0.751, p = 0.01) and fumarate (0.871, p = 0.01) and a moderate positive correlation with succinate (0.562, p = 0.01). HIF1α also showed a strong positive correlation with Notch 2, Notch 4, Ikaros, p53, Snail 1, CD-11d, TNFα, CCAR1, SIRT1, Foxo-3a, HSF1, IL-1β, UQCR2, and PSMB6 (1.00, p = 0.001) and a strong negative correlation with Notch1, Lgd, CDH1, GAPDH, UBQLN2, RPS18/18a, and PGGT1B (-1.00, p = 0.001), as shown in **Figures 7A–G**. All the Pearson correlations of HIF1α showed strong agreement with all of the above results.

### Biomarker Analysis by AUROC in CML Patients

**Figure 8:**
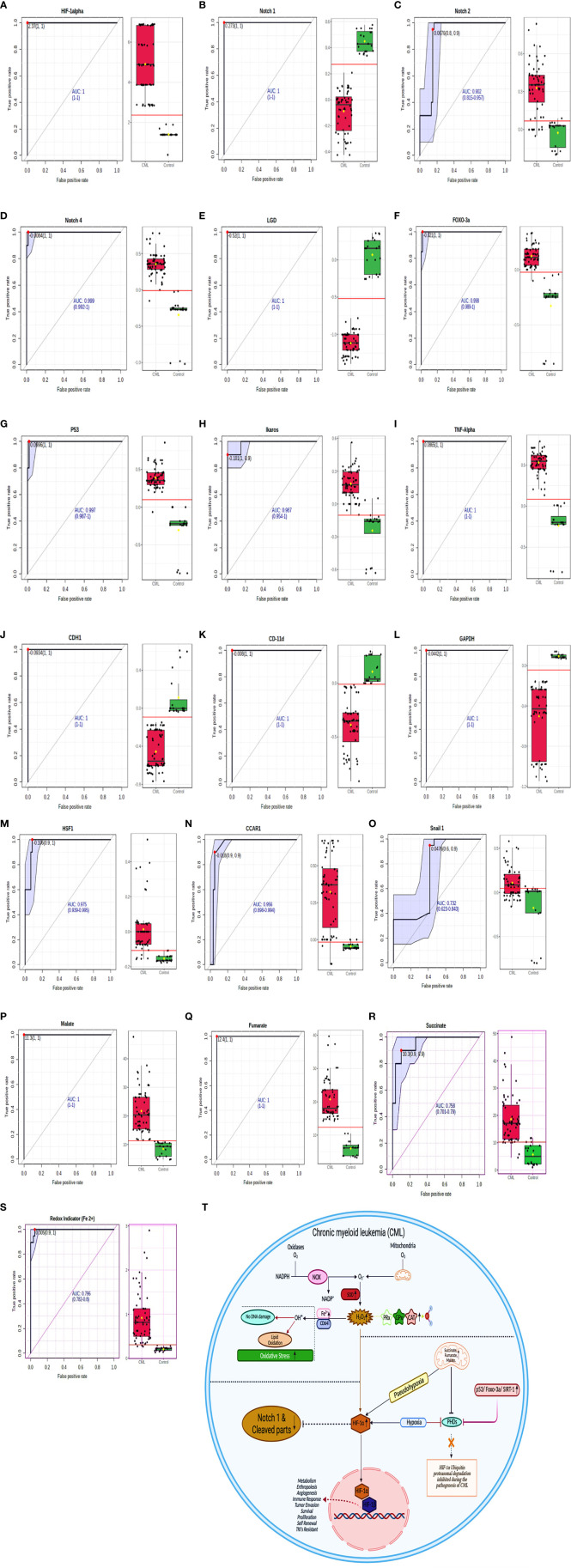
ROC for all the factors in CML samples. **(A–S)** AUROC shows that HIF1α, Notch 1, Notch 4, Lgd, Foxo-3a, p53, TNFα, CDH1, CD11d, GAPDH, malate, and fumarate have 100% sensitivity and 100% specificity, while Notch 2 (sensitivity- 80%, specificity- 90%), Ikaros, (sensitivity- 100%, specificity- 90%), HSF1 (sensitivity- 90%, specificity- 100%), CCAR1 (sensitivity- 90%, specificity- 90%), Snail1 (sensitivity- 60%, specificity- 90%), succinate (sensitivity- 90%, specificity- 90%), and Redox indicator Fe^2+^ (sensitivity- 90%, specificity- 100%). See also **Figure S2**. **(T)** Overall mechanism explained in HIF1α/Notch1 pathway.

To determine the clinical association of redox factors, Krebs oncometabolites, and all genes (in serum, plasma, blood lysates, bone marrow tissues) in the pathogenesis of CML, their diagnostic relevance was determined by the area under the receiver operating characteristic (AUROC) curve, and the operational cutoff value was defined. The analysis of AUROC showed that HIF1a, Notch 1, Notch 4, Lgd, Foxo-3a, p53, TNFa, CDH1, CD11d, GAPDH, Malate, and Fumarate had 100%, sensitivity and 100% specificity, while Notch 2 (sensitivity-80%, specificity- 90%), Ikaros, (sensitivity- 100%, specificity- 90%), HSF1 (sensitivity- 90%, specificity- 100%), CCAR1 (sensitivity- 90%, specificity- 90%), Snail1 (sensitivity- 60%, specificity- 90%), Succinate (sensitivity- 90%, specificity- 90%), and Redox indicator Fe2+ (sensitivity- 90%, specificity- 100%) were significantly associated in the blast crisis phase and exhibited a relatively high distinction power to serve as biomarkers in CML patients as shown in **Figures 8A–T**, extended **Figure S2**, quantitative details given in **Supplementary Table S6**.

## Discussion

Blast crisis is a terminal stage of chronic myeloid leukemia (CML), a clonal myeloproliferative disorder of hematopoietic stem cells distinguished into three phases: chronic, accelerated phases, and blast crisis (58, 59). Blast crisis withstands only a few months and is identified by the myeloid population’s expeditious growth and lymphoid differentiation apprehended blast cells (60). CML is consistently related to an acquired genetic aberration in the Philadelphia chromosome (Ph1), a shortened chromosome 22 resulting from reciprocal translocation of the long terms of chromosomes 9 and 22 (61, 62). This translocation gives rise to the BCR/ABL fusion gene, which consequentially translates into the p210^BCR/ABL^ oncoprotein (63, 64) in CML patients. The Leishman staining and flow cytometry results show the presence of 80% blast crisis cells in CML patients. However, the BCR/ABL translocation results showed the maximum presence of p210 and p190 with an overall coverage of b3a3, b2a3, and e1a3 translocations in all CML patients. These two translocations (p210 and p190) play a crucial role in leukemogenesis by altering the redox system, Krebs metabolites, hypoxia, and their associated genes. However, continuous oxidative stress in leukemic cells might be an interesting therapeutic target, and the possibility of developing redox chemotherapeutics has received much more attention in recent years. Recently, the practice of giving pro-oxidants in cancer therapy that selectively kill cancer cells through reactive oxygen production (ROS) in augmentation with conventional chemotherapy has been in use (65). The results of our study draw attention to the synergistic role of oxidants and antioxidants in the pathogenesis of BCR/ABL translocated cells. After exploring redox parameters, oxidative parameters, including CD64, Fe^2+^ redox indicator, ROS production, total nitric oxide, nitrite, nitrate, superoxide ion, MDA (lipid peroxidation), and protein carbonyl, were significantly elevated in almost 90% of CML cases in comparison to healthy controls. The CD64 marker was significantly high, which consequentially increased oxidative stress in blast crisis cells and was also responsible for releasing cytokines and reactive oxygen intermediates in myeloid cells. Antioxidant factors such as total antioxidant, glutathione peroxidase/reductase, catalase, and superoxide dismutase (SOD) levels were also found to be higher in CML cases. An increase in the antioxidant system decreases the level of vanin-1 proteins in CML cases. Vanin-1 is a novel family member of ectoenzymes (66–68). Some studies have reported that vanin knockout mice are more resistant to drugs and against lethal doses of γ-irradiation. Following irradiation, the mice helped reduce the apoptotic response. This protection is related to changes in the detoxifying potential of vanin 1 knockout tissues, characterized by elevated antioxidant levels (69). Our present study demonstrates that vanin-1 can be used as a prognostic biomarker in CML during treatment. Herein, we observed that an upregulated antioxidative system prevents DNA fragmentation and maintains integrity from elevated ROS levels in blast crisis cells of CML, whereas elevated ROS can only change the morphological behavior of cells through lipid peroxidation and protein carbonyl generation. Therefore, our findings disagree with pro-oxidant therapy along with chemotherapy, as mentioned in previous reports, as elevated antioxidant parameters counterbalance ROS generation in CML. Compared with normal cells, leukemia cells encounter more oxidative stress, and increased ROS production mainly results from inefficient mitochondrial respiration (70). However, excessive ROS production can induce HIF1α stabilization, which promotes glycolysis to attenuate oxidative phosphorylation of the PHD domain and ROS generation in the classical stimulus-response loop to maintain redox homeostasis (71, 72), which is in agreement with our study. HIF1α can trigger more than thousands of genes that help in cancer metastasis. Three different pathways are involved in HIF1α stabilization by inhibiting the PHD domain. The first pathway, which helps stabilize HIF1α, is Krebs cycle metabolites (succinate, fumarate, and malate). The levels of succinate, fumarate, and malate were significantly higher in almost 98% of CML cases than in healthy controls. These metabolites are involved in promoting the accumulation and stabilization of HIF1α in the presence of oxygen and create a pseudohypoxic environment through inhibition of the PHD domain (29, 32). Although previous studies have shown that endogenous fumarate inhibits GAPDH through the succination process (73), GAPDH has not to be used as an internal control because its expression is lower in CML cells. HIF1α also induces the production of inflammatory factor-like TNFα and IL-1β responsible for metastasis (18), and our result also follows the same trajectory.

The second mechanism involves the regulation of HIF1α through the ubiquitin proteasomal pathway. Under normoxic conditions, the von Hippel Lindau-mediated ubiquitin-proteasome pathway rapidly degrades HIF1α (25, 74). The LC/MS and RT-PCR results confirmed that all the factors associated with the ubiquitin proteasomal pathway (WWP, M3K14, TRI22, PPIL2, M3K15, etc.) and apoptosis factors (CAR14, APAF, CARD1, CAR11, DEDD2, CFLAR, etc.) expression is decreased in CML patients compared with healthy controls, while the anti-apoptotic protein survivin protein (BIRC2) expression increases in blast crisis cells, so the overall findings reveal that there is no anoikis in CML cells. The third pathway involves HIF1α stabilization and regulation by an oxygen-independent modulator, which includes SIRT1, Foxo-3a, p53, and HSF1 (75). The overexpression of SIRT1 promotes mitochondrial biogenesis by deacetylation, which results in the activation of HIF1α (76). Upregulation of SIRT1 has been implicated in the prevention of premature cellular senescence and pathogenesis of chronic diseases (77–79). SIRT1 mRNA transcription is also coupled with hypoxia, whereas the feedback control mechanism regulates the levels of the SIRT1 transcript by the p53/forkhead box O-3a (Foxo-3a)/SIRT1 pathway (80). However, our results show that all these factors are upregulated in CML cases with many folds and help in stabilizing HIF1α, which participates in the progression of CML. A variety of mutations have been associated with the progression of blast crisis cells, including the BCR/ABL, p53, and Ikaros (IKZF1) genes. IKZF1 mutation has been identified in the activation of several recurrent genes that help in the metastasis of cancer cells in a hypoxic environment (81), which can be detected in both myeloid and lymphoid BC (82, 83). BC cells here show higher expression of IKZF1 in CML patients. We next performed an association study to examine the relationship between HIF1α and notch1. Previous studies indicated that two mechanisms are associated with crosstalk between HIF and Notch. The first involves HIF1α binding to cleaved Notch1, which results in stabilization and activation of the Notch signaling pathway (84). The second describes HIF1α as a repressor of the Notch1-hes family b-HLH transcription factor 1 (HES1) negative feedback loop by inhibiting the binding of HES1 to their promoter, resulting in enhanced Notch signaling (85). However, a precise relationship between HIF and Notch has not yet been established, specifically in CML cells. Our finding does not follow the same tangent as explained in previous reports that HIF1α activates the notch1-signaling pathway. Conversely, our observation suggests that upregulated HIF1α suppresses the Notch1 signaling pathway (MAML1, ADAM, ASAP3, ARAP1, etc.) in CML patients with blast crisis cells, whereas Notch2 and Notch4 were upregulated, which is in agreement with previous findings. In addition, we explored the role of the lethal giant disc (Lgd) in tumor suppression and its association with Notch1 in CML cells. Some reports have indicated that Lgd is a novel, conserved C2-domain protein that regulates Notch receptor endosomal trafficking (86). There is increasing evidence that endosomal pathways play an important role in the regulation and activation of Notch signaling (87), and the tumor suppressor gene lgd (lethal giant disc) is a regulator of the activity of the Notch pathway during wing and brittle development in Drosophila (88). Lethal giant disc (Lgd), which might act as a tumor suppressor along with notch1, in CML patients we found that Lgd and Notch 1expression was significantly downregulated with several folds and behaves like a tumor suppressor. Finally, the study was carried out to establish a protein-protein interaction where we found that HIF1α is a master regulator of all the genes studied in CML, which provides insight into the involvement of all the genes directly or indirectly through a common axis, i.e., the HIF1α gene. In the Pearson correlation analysis between all the factors, we found that HIF1α showed a positive correlation (directly related) with ROS, total NO, nitrite, nitrate, superoxide ion, malate, fumarate, Notch 2, Notch 4, Ikaros, p53, Snail 1, CD-11d, TNFα, CCAR1, SIRT1, Foxo-3a, HSF1, IL-1β, UQCR2, and PSMB6, a moderate positive correlation with succinate, and a strong negative correlation (inversely related) with Notch1, Lgd, CDH1, GAPDH, UBQLN2, RPS18/18a, and PGGT1B. By ROC curve analysis, we evaluated the diagnostic value of all the factors in CML patients and found that HIF1α, Notch 1, and other genes might be strong candidates for use as diagnostic markers for CML patients.

## Conclusions

This study revealed that HIF1α acts as a master regulator in the pathogenesis of leukemia and might be used, as a therapeutic target for CML. Pro-oxidant therapy practice might not help kill cancer cells and prevent pathogenesis in CML cells. Notch1 plays an antagonist role with HIF1α and acts as a tumor suppressor in CML patients, which needs further elucidation. Notch1 and HIF1α, along with other genes, will open a set of novel biomarkers for the detection of CML.

## Data Availability Statement

The original contributions presented in the study are included in the article/**Supplementary Material**. Further inquiries can be directed to the corresponding author.

## Ethics Statement

The studies involving human participants were reviewed and approved by KGMU. Written informed consent to participate in this study was provided by the participants’ legal guardian/next of kin.

## Author Contributions

VS designed the research, performed the experiments, and acquired and analyzed the data; RK and AT performed clinical diagnosis; VS and RS drafted the article; RS and AM revised the manuscript; VS and RS performed the statistical analysis and conceived the grants. All authors contributed to the article and approved the submitted version.

## Funding

The work was supported by Department of Biotechnology (BT/IN/Indo-US/Foldscope/39/2015) and ICMR (45/3/2019-Hae/BMS), and (56/6/2019-HAE/BMS) India. The English editing was done by AJE.

## Conflict of Interest

The authors declare that the research was conducted in the absence of any commercial or financial relationships that could be construed as a potential conflict of interest.

## Publisher’s Note

All claims expressed in this article are solely those of the authors and do not necessarily represent those of their affiliated organizations, or those of the publisher, the editors and the reviewers. Any product that may be evaluated in this article, or claim that may be made by its manufacturer, is not guaranteed or endorsed by the publisher.
