# Supplementary material for: The Molecular Role of HIF1α Is Elucidated in Chronic Myeloid Leukemia
Source: Front Oncol. 2022 Jun 30;12:912942. doi: 10.3389/fonc.2022.912942 (PMC9279726; doi:10.3389/fonc.2022.912942)
Supplement: Supplementary file 1 [file DataSheet_1.docx]

Supplemental Information

**The molecular role of HIF1α is elucidated in chronic myeloid leukemia.**

Vivek Singh^1^, Ranjana Singh^1, *^, Rashmi Kushwaha^2^, Shailendra Prasad Verma^3^, Anil Kumar Tripathi^3^, Abbas Ali Mahdi^1^

^1^Department of Biochemistry, King George’s Medical University, Lucknow, U.P., India,

^2^Department of Pathology, King George’s Medical University, Lucknow, U.P., India,

^3^Department of Clinical Hematology, King George’s Medical University, Lucknow, U.P., India,

^*^Correspondence to; Dr. Ranjana Singh, Additional Professor, Department of Biochemistry, King George’s Medical University, Lucknow, U.P., India- 226003. Email: [ranjanasingh.2509@rediffmail.com/](mailto:ranjanasingh.2509@rediffmail.com/) ranjanasingh@kgmcindia.edu

Supplemental Figures…………………………………………………………………………………… 2

Figure S1: Complete Flow result of CML patient……………………………………………………… 2-4

Figure S2: Biomarker Analysis by ROC curve………………………………………………............... 5-15

Supplemental Tables……………………………………………………………………………………. 16

Table S1: All the primers details used in RT-PCR……………………………………………………... 16

Table S2: Details of LCMSMS Flow Rate: 0.3mL/min………………………………………………... 17

Table S3: Overview of samples and clinical data of the patients………………………………………. 17

Table S4: All mathematical the details of protein array (LCMSMS)………………………………….. 17

Table S5: String quantitative details of protein-protein interaction…………………………………… 17

Table S6: Quantitative details of ROC curve analysis………………………………………………… 17-18

Supplemental Figures


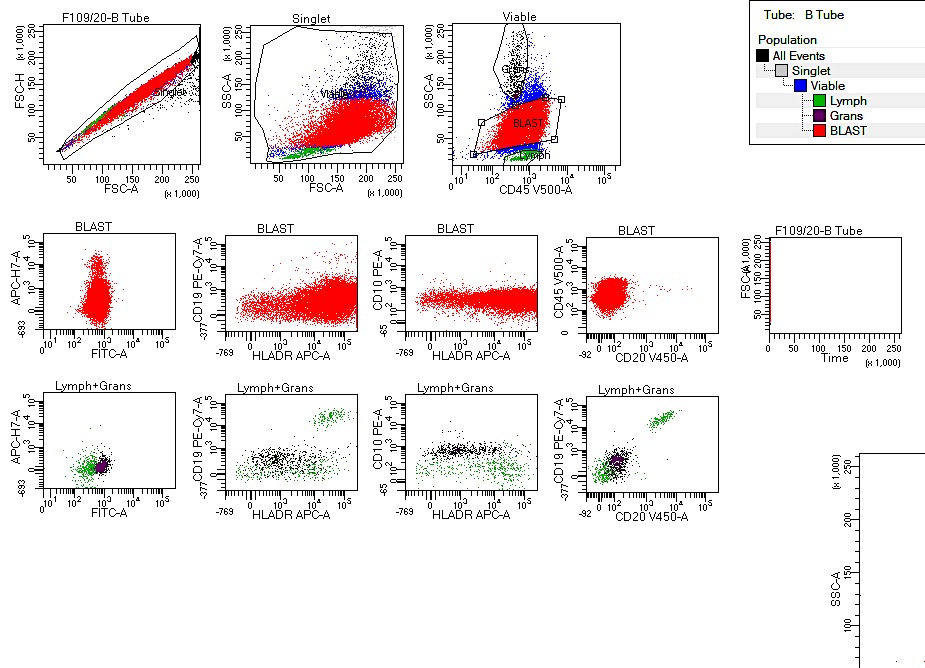


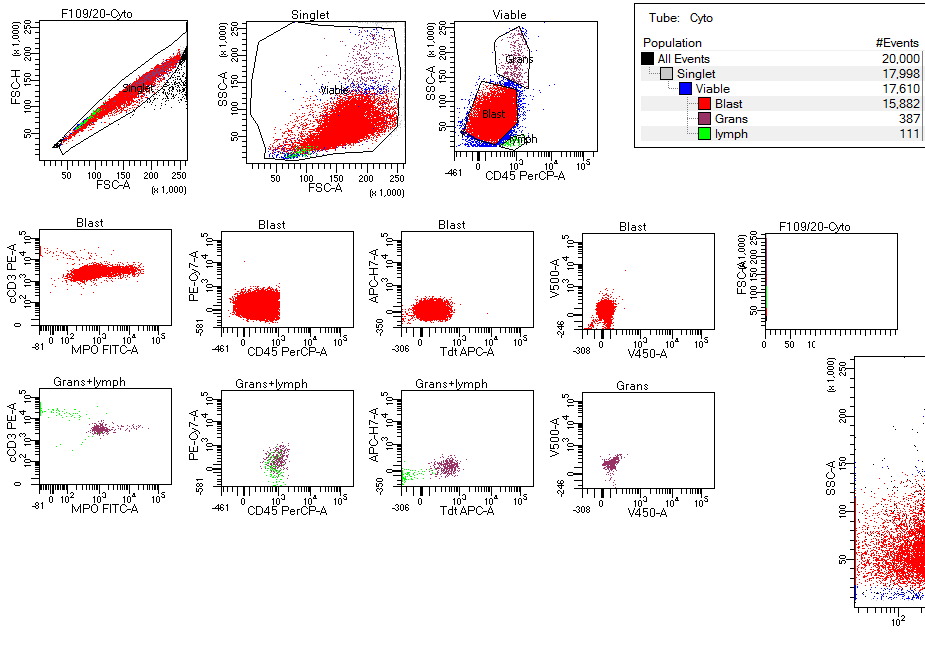


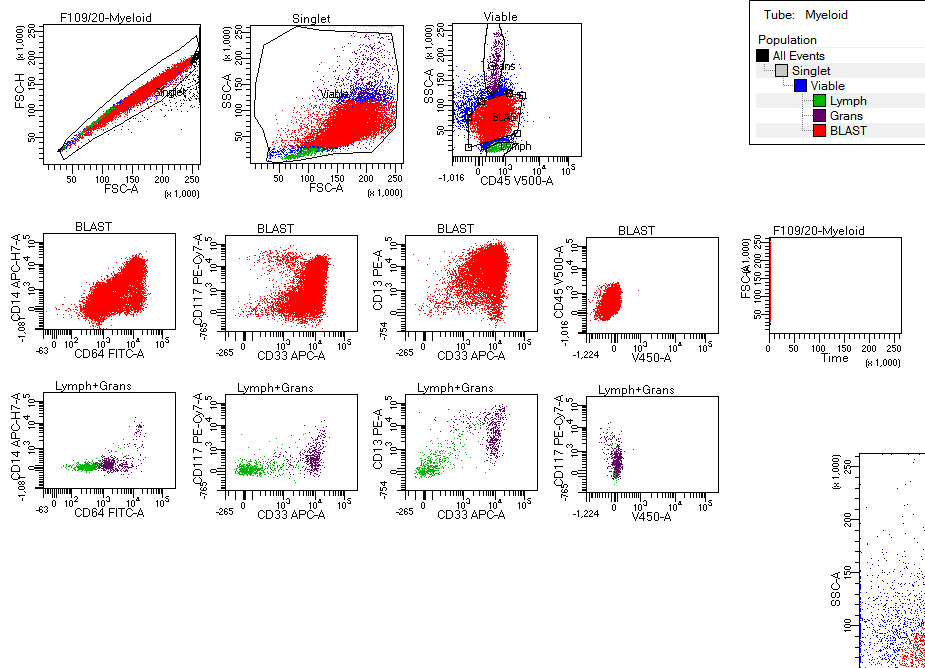


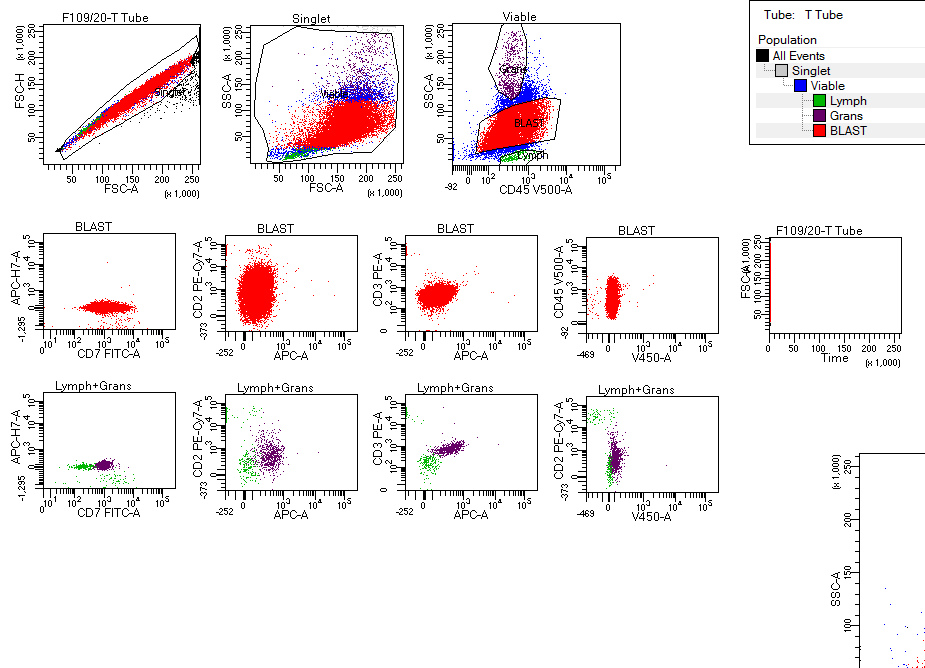


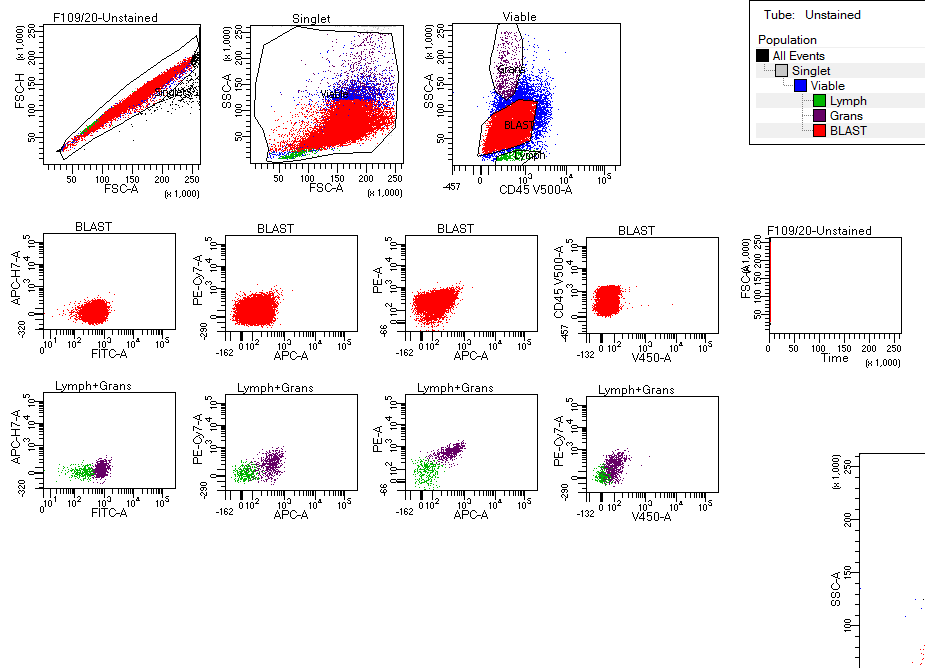


**Figure S1: Complete Flow result of CML patient.** All the linage markers show in this figure.

A.

B.


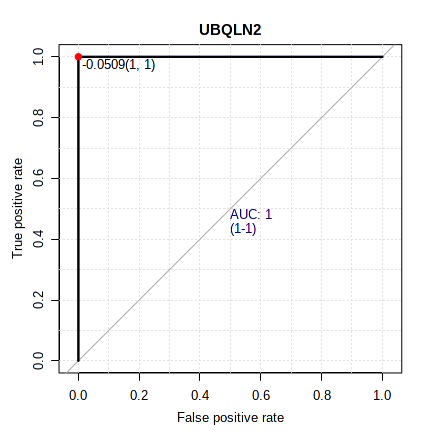

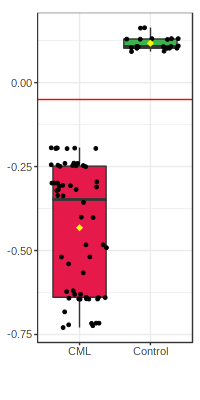

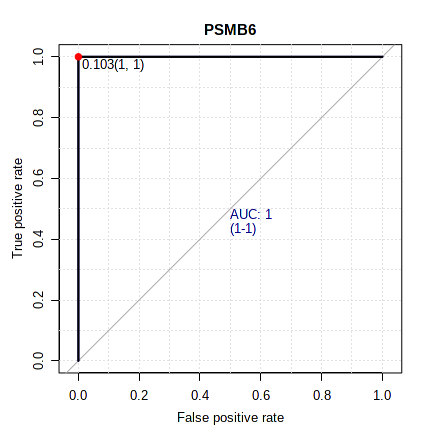

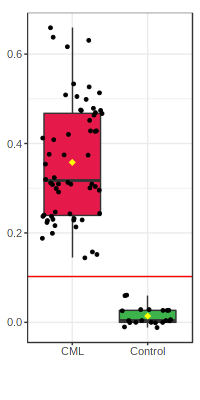


C.


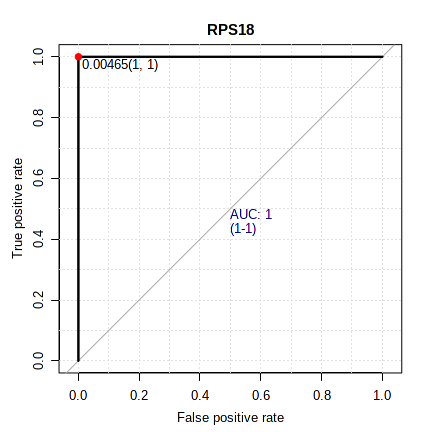

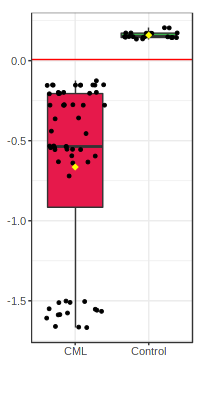


D.


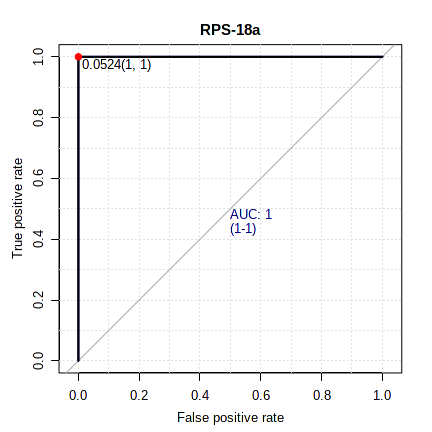

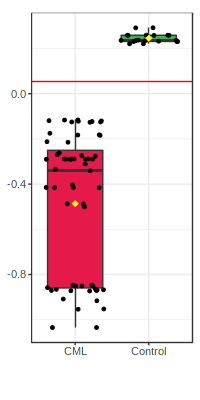


E.


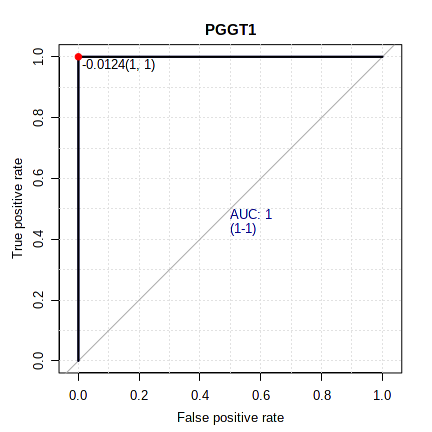

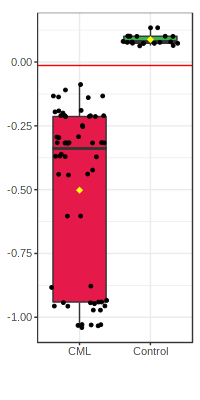


F.


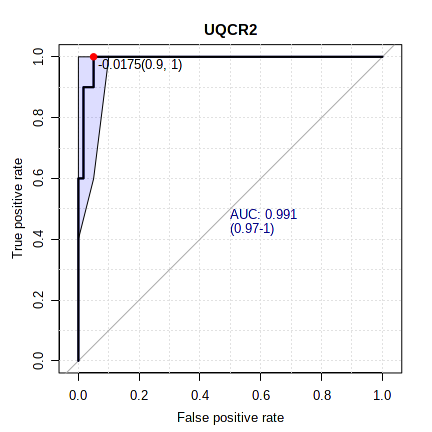

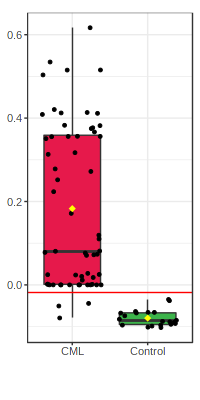


G.


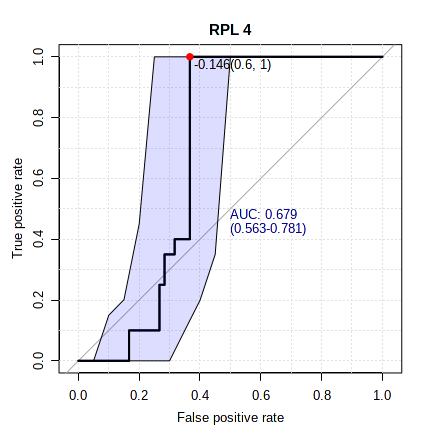

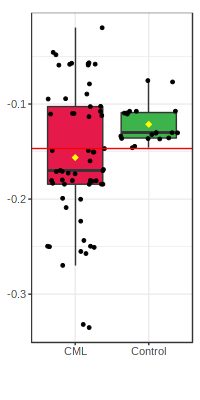


H.


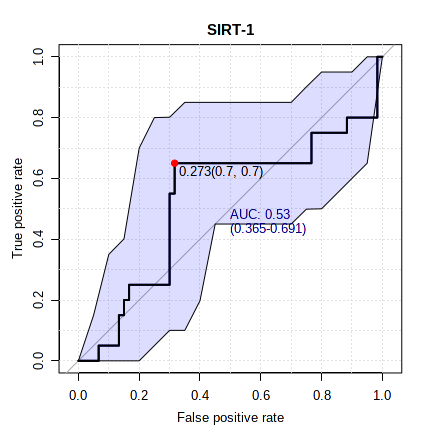

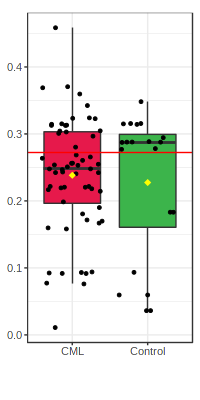


I.


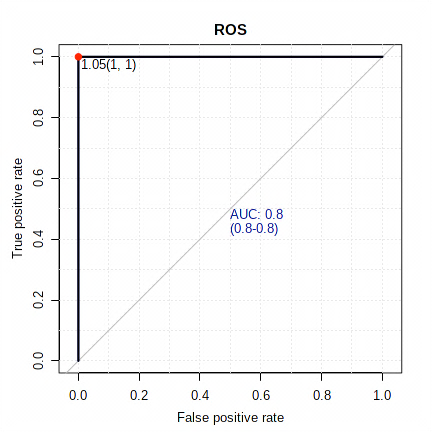

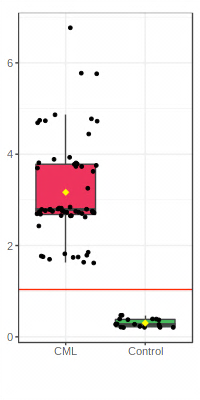


J.


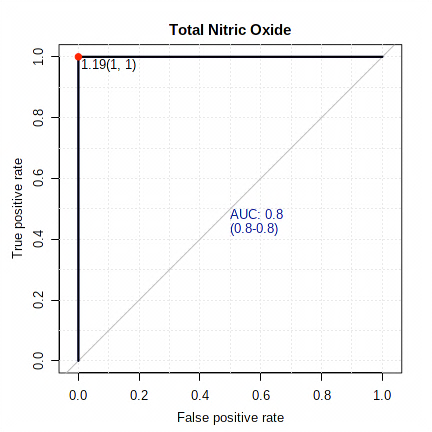

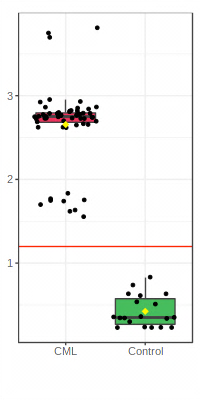


K.


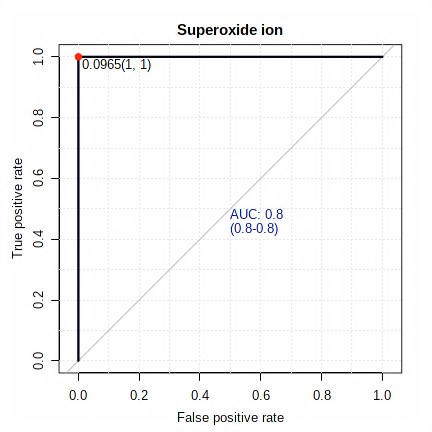

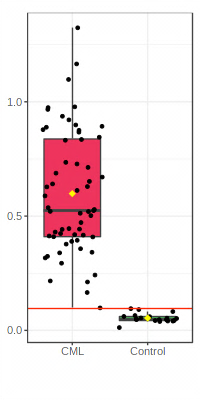


L.


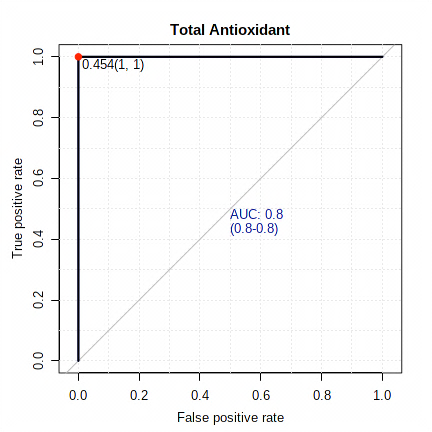

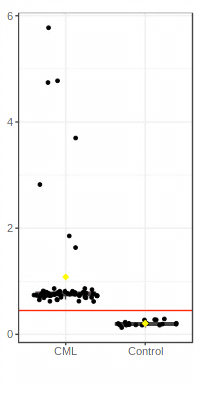


M.


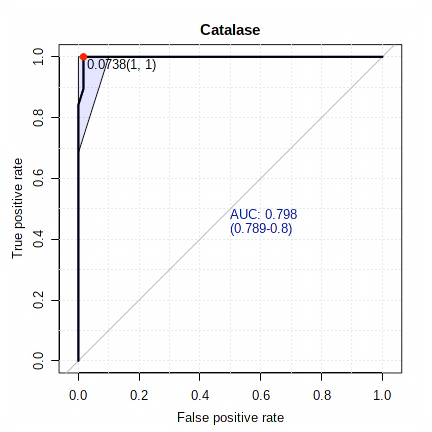

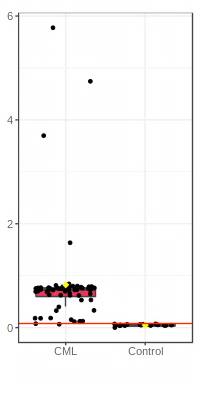


N.


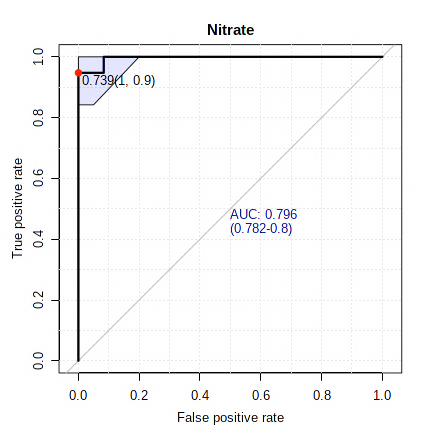

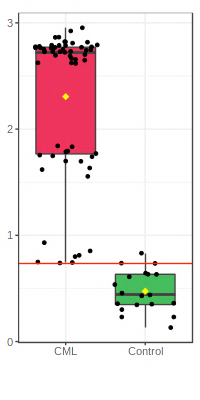


O.


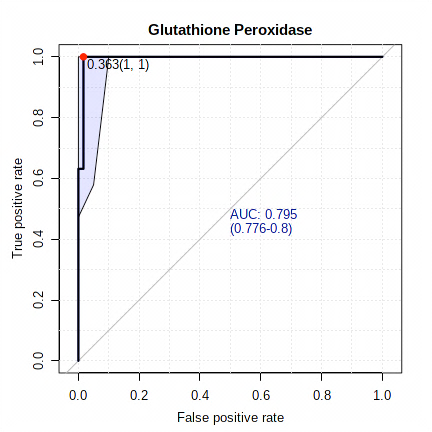

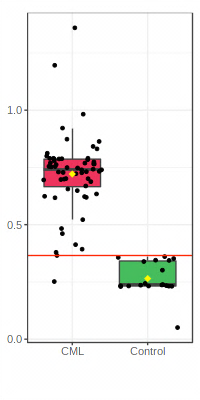


P.


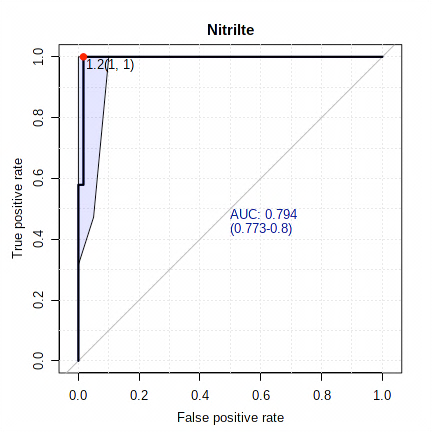

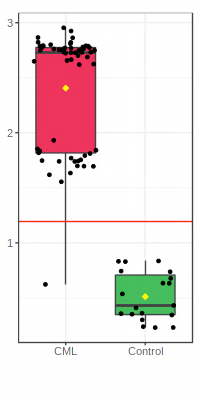


Q.


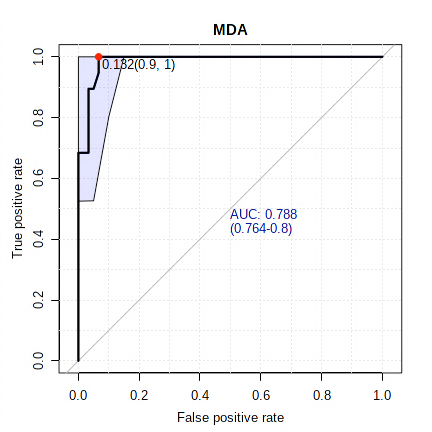

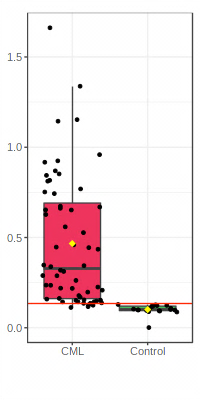


R.


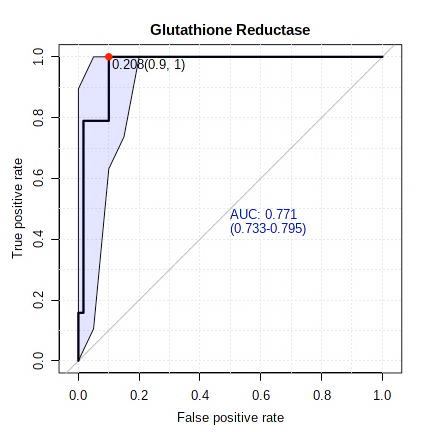

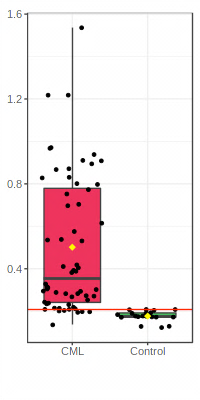


S.


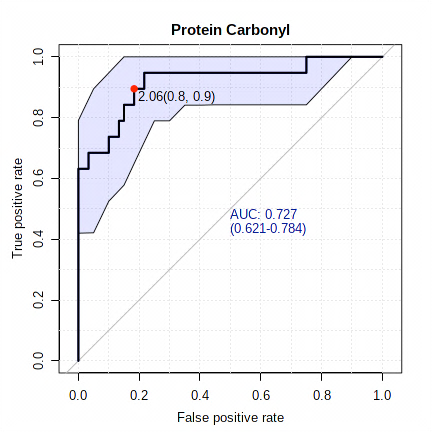

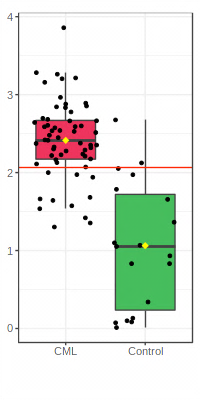


T.


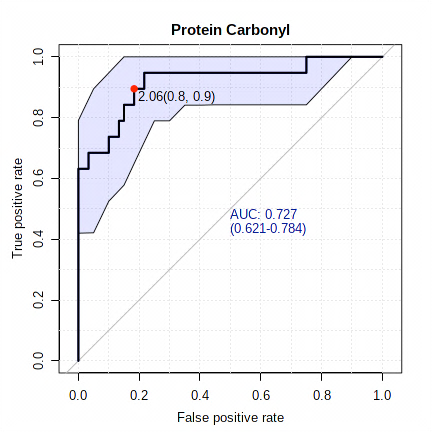

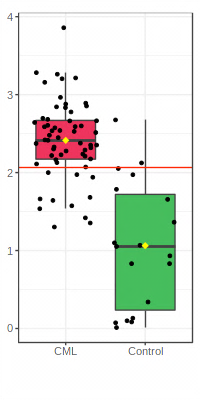


U.


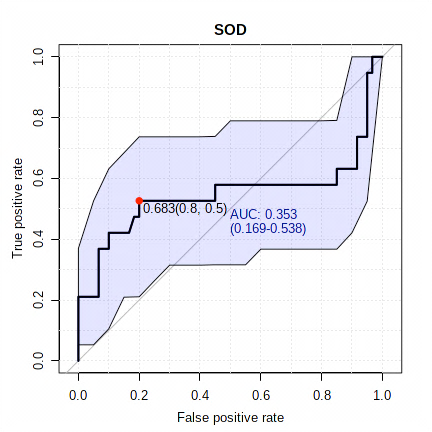

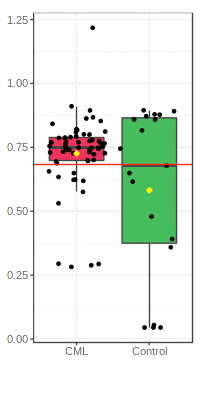


**Figure S2: Biomarker Analysis by ROC curve.** (A-U) figure shows that ROC curve with operational cutoff, sensitivity, and specificity at top of left and AUC at the diagonal lines with confidence intervals (CI).

Supplemental Tables

**Table S1: All the primers details used in RT-PCR.**

| S.No. | Genes | Forward primer | Reverse Primer | Annealing Temp (^o^C) | Amplicon Length |
| --- | --- | --- | --- | --- | --- |
| 1. | β- Actin | TCACAATGTGGCCGAGGACTTTT | AGAAGTGGGGTGGCTTTTAGGATG | 60 | 115 |
| 2. | CCAR1 | AGCTGCATTACAACAGCAATA | GGCTGTGTTAAGAGGGTTTG | 60 | 96 |
| 3. | Notch 1 | AACAACAGGGAGGAGACA | GGTCCATATGATCCGTGATG | 60 | 112 |
| 4. | Notch 2 | AAGGGCTTTAAAGGCTATAACT | TGTCATCAAAGCAGGTTCC | 60 | 88 |
| 5. | Notch 4 | GAGGGTCCACGTTGTGAG | TCTGGGCCTATGAAACCTG | 60 | 137 |
| 6. | UBQLN2 | CAGCCTGAAGGATCAGTGTAGT | AGGGTCTCTTTATGGGAGAAGC | 60 | 84 |
| 7. | RPL-4 | GTGGGACGTTTCTGCATTTG | TGTGCATGGGAAGATTGTAGT | 60 | 112 |
| 8. | Ikaros | CGAATACTCTGTCTGCCCTATC | CCCATCTCTTCTCCCTCTCATA | 60 | 122 |
| 9. | RPS-18 | CAGCCAGGTCCTAGCCAATG | CCATCTATGGGCCCGAATCT | 60 | 82 |
| 10. | RPS18-a | AATCCACGCCAGTACAAAGATCCCA | TTTCTTCTTGGACACACCCACGGT | 60 | 241 |
| 11. | UQCR2 | CCTGCGGGGTGATGTTGATA | CAGCTACTTCCCAACGACCGA | 60 | 83 |
| 12. | PSMB6 | TGACACCTATTCACGACCGC | GGACCAGTGGAGGCTCATC | 60 | 129 |
| 13. | PGGT1B | CTGTGTTTCCGAGGCTCTT | GCATGAGAGGCCAGTGTAGG | 60 | 118 |
| 14. | TNF-α | CCCTCACACTCAGATCATCTTCT | GCTACGACGTGGGCTACAG | 60 | 88 |
| 15. | p53 | GCCTCTTTCCTAGCACTGCCCAAC | CCCAAGACTTAGTACCTGAAGGGT | 60 | 102 |
| 16. | GAPDH | CAAGGTCATCCATGACAACTTTG | GGGCCATCCACAGTCTCCTG | 60 | 91 |
| 17. | IL-1β | GCAACTGTTCCTGAACT | ATCTTTTGGGGTCCGTCAA | 60 | 112 |
| 18. | CD-11d (ITGAD) | CCAGGCAATGGAGGAAGAA | CTCGGGTCTGCTCATAGTAATG | 60 | 147 |
| 19. | SIRT1 | AGAACCCATGGAGGATGAAAG | TCATCTCCATCAGTCCCAAATC | 60 | 111 |
| 20. | Foxo-3a | CCACCCTTGGCCTCTAAATAA | GAGAACAGCAGATCCCAAGAG | 60 | 114 |
| 21. | HSF1 | TGGTCAAGCCAGAGAGAGA | CACACTGGTCACTTTCCTCTT | 60 | 101 |
| 22. | HIF1 | CCAGTTACGTTCCTTCGATCAG | GTAGTGGTGGCATTAGCAGTAG | 60 | 135 |
| 23. | Lgd | TCAAGGATGTTCTCCAGAAAG | ATCGTCCTCACCATATCCA | 60 | 123 |
| 24. | CDH 1 | GAACAGCACGTACACAGCCCT | GCAGAACTGTCCCTGTCCCAG | 60 | 89 |
| 25. | Snail 1 | TGCAGGACTCTAATCCAAGTTTAC | GTGGGATGGCTGCCAGC | 60 | 118 |

**Table S2: Details of LCMSMS Flow Rate: 0.3mL/min**

| S.No. | Time | Buffer A  (0.1% FA in water) | Buffer B  (0.1% FA in ACN) |
| --- | --- | --- | --- |
| 1. | 1 | 98 | 2 |
| 2. | 30 | 50 | 50 |
| 3. | 40 | 20 | 80 |
| 4. | 50 | 98 | 2 |

Instruments Used: Q-TOF SYNAPT G2 MASS SPECTROMETRY with ESI Source and UPLC

**Table S3: Overview of samples and clinical data of the patients.**

This table is provided as separate Excel file.

**Table S4: All mathematical the details of protein array (LCMSMS).**

This table is provided as separate Excel file.

**Table S5: String quantitative details of protein-protein interaction.**

This table is provided as separate Excel file.

**Table S6: Quantitative details of ROC curve analysis.**

| S.No. | Factors | AUROC | p value | Fold Change  Log2 FC  (Control/CML) |
| --- | --- | --- | --- | --- |
| 1. | ROS (****) | 1.0 | 1.0056E-17 | 3.388 |
| 2. | Total Nitric Oxide (****) | 1.0 | 8.22E-33 | 2.647 |
| 3. | Superoxide ion (****) | 1.0 | 3.08E-13 | 3.4764 |
| 4. | Total Antioxidant (****) | 1.0 | 5.808E-4 | 2.3903 |
| 5. | Catalase (****) | 0.99781 | 7.92E-4 | 4.09 |
| 6. | Redox indicator (Fe2+) (****) | 0.99561 | 8.68E-7 | 2.1482 |
| 7. | Nitrate (****) | 0.99561 | 5.281E-18 | 2.2832 |
| 8. | Glutathione Peroxidase (****) | 0.99386 | 1.1408E-17 | 1.4467 |
| 9. | Nitrite (****) | 0.99298 | 1.13E-24 | 2.2332 |
| 10. | MDA (****) | 0.9864 | 2.74E-5 | 2.2077 |
| 11. | Glutathione Reductase (****) | 0.96842 | 4.1E-5 | 1.5049 |
| 12. | Protein carbonyl (****) | 0.91754 | 5.553E-13 | 1.184 |
| 13. | ROS (DCFDA) (****) | 0.81711 | 2.2599E-4 | 1.2273 |
| 14. | SOD (**) | 0.54868 | 0.009 | 0.31991 |
| 15. | Malate (****) | 1.0 | 6.8559E-11 | 1.3609 |
| 16. | Fumarate (****) | 1.0 | 1.0292E-16 | 1.7905 |
| 17. | Succinate (****) | 0.9533 | 1.6415E-8 | 1.7231 |
| 18. | HIF1α (****) | 1.0 | 3.0157E-10 | 1.8007 |
| 19. | Notch1 (****) | 1.0 | 3.7079E-23 | -0.95244 |
| 20. | CDH1 (****) | 1.0 | 7.77E-17 | -1.2873 |
| 21. | CD11d (****) | 1.0 | 1.3135E-13 | -0.82285 |
| 22. | TNFα (****) | 1.0 | 9.3215E-28 | 3.2131 |
| 23. | Lgd (****) | 1.0 | 1.696E-41 | -3.3169 |
| 24. | UBQLN2 (****) | 1.0 | 4.282E-21 | -0.97842 |
| 25. | PSMB6 (****) | 1.0 | 1.265E-18 | 1.9391 |
| 26. | RPS18 (****) | 1.0 | 4.0722E-9 | -1.3097 |
| 27. | RPS18a (****) | 1.0 | 2.981E-16 | -1.4092 |
| 28. | PGGT1B (****) | 1.0 | 2.243E-11 | -0.89755 |
| 29. | GAPDH (****) | 1.0 | 7.8022E-12 | -0.84476 |
| 30. | Notch 4 (****) | 0.99833 | 1.9354E-21 | 2.9638 |
| 31. | Foxo-3a (****) | 0.9975 | 1.02E-17 | 2.1207 |
| 32. | p53 (****) | 0.99667 | 8.748E-23 | 2.8633 |
| 33. | UQCR2 (****) | 0.99 | 5.034E-8 | 1.8107 |
| 34. | Ikaros (****) | 0.985 | 3.07E-16 | 2.1033 |
| 35. | HSF1 (****) | 0.97167 | 9.253E-7 | 1.4555 |
| 36. | CCAR1 (****) | 0.95333 | 2.719E-9 | 2.1591 |
| 37. | Notch 2 (****) | 0.89583 | 1.01E-10 | 2.092 |
| 38. | Snail1 (****) | 0.73083 | 2.8611E-6 | 1.5436 |
| 39. | RPL4 (*) | 0.678 | 0.03 | 0.6395 |
| 40. | SIRT-1 (ns) | 0.5283 | 0.64786 | 0.78546 |
